# Wearable Inertial Sensors to Assess Standing Balance: A Systematic Review

**DOI:** 10.3390/s19194075

**Published:** 2019-09-20

**Authors:** Marco Ghislieri, Laura Gastaldi, Stefano Pastorelli, Shigeru Tadano, Valentina Agostini

**Affiliations:** 1 Department of Electronics and Telecommunications, Politecnico di Torino, 10129 Torino, Italy; 2 Department of Mathematical Sciences, Politecnico di Torino, 10129 Torino, Italy; 3 Department of Mechanical and Aerospace Engineering, Politecnico di Torino, 10129 Torino, Italy; stefano.pastorelli@polito.it; 4 National Institute of Technology, Hakodate College, Hakodatate 042-8501, Japan; tadano@eng.hokudai.ac.jp; 5 Graduate School of Health Science, Hokkaido University, Sapporo 060-0808, Japan

**Keywords:** postural sway, postural balance, posturography, IMU, inertial sensors, wearable, accelerometers, validation, Parkinson’s disease, fall risk

## Abstract

Wearable sensors are de facto revolutionizing the assessment of standing balance. The aim of this work is to review the state-of-the-art literature that adopts this new posturographic paradigm, i.e., to analyse human postural sway through inertial sensors directly worn on the subject body. After a systematic search on PubMed and Scopus databases, two raters evaluated the quality of 73 full-text articles, selecting 47 high-quality contributions. A good inter-rater reliability was obtained (Cohen’s kappa = 0.79). This selection of papers was used to summarize the available knowledge on the types of sensors used and their positioning, the data acquisition protocols and the main applications in this field (e.g., “active aging”, biofeedback-based rehabilitation for fall prevention, and the management of Parkinson’s disease and other balance-related pathologies), as well as the most adopted outcome measures. A critical discussion on the validation of wearable systems against gold standards is also presented.

## 1. Introduction

Human balance in the upright stance can be quantitatively evaluated by means of a posturographic examination. Posturography is the systematic measurement and interpretation of quantities that characterize postural sway in upright stance. In the clinical field, posturography is used to estimate fall risk in geriatric subjects [1] and to objectively evaluate balance-related disabilities (such as Parkinson’s disease, concussion, and stroke) and rehabilitation protocols [2,3,4,5], while in sport science, posturography is used to appraise subtle differences in the balance performances of athletes [6]. The increasing interest towards the study of balance has led to a continuous evolution of the methods used to carry out this examination. Traditionally, posturography exploits a force plate to evaluate the body’s postural sway by recording the trajectory of the Center of Pressure (COP), which is the point of application of the resultant ground reaction force [7]. Although the force plate is considered the gold standard to obtain reliable balance measurements, it is expensive and heavy to transport, making it impractical in clinical settings and sport centers. In recent years, wearable sensors based on miniaturized Inertial Measurement Units (IMUs) or Magneto Inertial Measurement Units (MIMUs) are increasingly being used in posturography, as demonstrated by the high number of papers focusing on this topic [8,9,10,11,12,13,14]. Subjects can easily wear these sensors on various body segments, through elastic belts or Velcro^®^ bands. The number of sensors and their positioning generally depend on the application considered. 

A wearable inertial sensing unit typically includes accelerometers, gyroscopes, and magnetometers. A triaxial accelerometer measures the proper linear acceleration of movements in a sensor-fixed three-dimensional (3D) frame; measured data include both motion and gravity components. A triaxial gyroscope measures its proper angular velocity in a 3D space, and the components of the rate of turn are assessed in a sensor-fixed three-dimensional frame; rotations around three orthogonal axes are commonly defined as Euler angles, e.g., “roll”, “pitch”, and “yaw”. A magnetometer measures both amplitude and direction of the local magnetic field in a 3D space; magnetic field components are stated in a sensor-fixed three-axes frame. Usually, accelerometer, gyroscope, and magnetometer measurements refer to a common three-axes frame fixed to the sensing IMU.

However, wearable sensors have not yet become a standard in posturography due to the unknown accuracy of IMU-based evaluations for balance assessment with respect to the gold standard force platform. If proven accurate, the use of wearable sensors for balance measurements would be ideal, since they are low cost and easily portable in different environments.

In the literature on balance control, fall risk assessment through wearable sensors is a debated topic [15,16,17,18,19,20,21,22,23]. Three systematic reviews specifically focused on the objective estimation of fall risk in geriatric populations: the first one, dating back to 2013, addressed the use of inertial sensors for fall risk assessment [19]; the second one, in 2017, addressed balance and fall risk assessments with mobile phone technology [20]; while the third one, in 2018, considered novel sensing technologies in fall risk assessment in older adults [21]. Another review provided insight into the detection of “near falls” (slips, trips, stumbles, and temporary loss of balance) using wearable devices [22]. An additional review targeted activity trackers for senior citizens [23] for monitoring various physical activity indicators and analyzed fall detection and prediction.

Among the various pathologies affecting balance performance, it is widely recognized that Parkinson’s Disease (PD) is a condition that may greatly benefit from an innovative clinical management of patients based on wearable monitoring technologies. A systematic review, published in 2013, discussed wearable technology and the principal postural parameters that should be analyzed for assessing PD [24]. Another systematic review, published in 2015, analyzed wearable sensor use for assessing both standing balance and walking stability in people with PD [25]. Finally, a systematic review, published in 2016, highlighted the characteristics and validity of monitoring technologies to assess PD [26]. Another commonly reported balance disorder that may strongly benefit from the use of wearable sensing technology is Multiple Sclerosis (MS). A recent systematic review, published in 2018, analyzed the validity of wearable sensor use for mobility and balance tracking in patients affected by MS [27]. Besides the constant need for rehabilitation professionals to have reliable balance outcome measures, there is a growing interest in the development of wearable systems specifically designed for the market of “active aging”. These systems may address both healthy and pathological populations. In this context, balance training based on wearable sensors and biofeedback constitutes a promising field of investigation. In 2016, a systematic review focused on balance improvement effects of biofeedback systems with wearable sensors [28]. In 2018, a systematic review and meta-analysis of randomized controlled trials analyzing both healthy and patient populations provided valuable knowledge on the effects of wearable sensor-based balance and gait training on balance, gait, and functional performance [29]. In addition, a review specifically analyzed smartphone applications to perform body balance assessments [30].

Despite the expanding body of evidence supporting the use of wearable sensors to assess postural balance, it is important to recognize that this area of research is still developing. As described before, several other systematic reviews have been published in the last years, focusing on postural balance assessment of sample populations affected by different balance-related pathologies. This study extends previous efforts by reviewing a large number of papers that use wearable sensors to assess postural balance and by providing a detailed overview of the most commonly reported applications that involve the use of wearable sensors to assess postural balance. The objectives of this work are (1) to select high-quality papers that adopt wearable inertial sensors for quantitatively evaluating standing balance; (2) to highlight the most important clinical applications in the framework of the fast-growing consumer market of IMUs, including rehabilitation and biofeedback; (3) to investigate the most common sensor placement and test protocols; (4) to describe the main parameters and outcome measures adopted; (5) to indicate which works perform a validation against a gold standard or a clinical score; and (6) to suggest future design directions of IMU-based wearable systems.

## 2. Methods

This review was performed according to the Preferred Reporting Items for Systematic Reviews and Meta-Analyses (PRISMA) statement [31].

### 2.1. Search Strategy

PubMed and Scopus electronic databases have been interrogated in February 2019 to identify articles measuring postural balance through IMU wearable sensors. The following keywords were used for the electronic database search within the title and/or the abstract: “posturography”, “postural sway”, “postural control”, “balance”, “IMU”, “MIMU”, “inertial sensor”, “accelerometer”, “sensor”, “wearable”, “smartphone”, and “activity tracker”. Specifically, the query that was used to search the articles in the databases was (“posturography” OR “postural sway” OR “postural control” OR “Balance”) AND (“IMU” OR “inertial measurement unit” OR “MIMU” OR “magneto inertial measurement unit” OR “inertial sensor” OR “accelerometer” OR “wearable sensor” OR “smartphone” OR “activity tracker”). In addition to the electronic database search, the reference lists of all the identified articles were searched by hand in order to identify additional relevant studies. The literature search was conducted by M.G.

### 2.2. Study Selection and Quality Assessment

After the initial electronic database search was completed, one rater (M.G.) screened the titles and the abstracts of each included article and decided on the suitability of the study for inclusion in this review. Articles were excluded if they (i) were not written in English, (ii) were an abstract and/or included in the proceedings of a conference, (iii) were a review article or a case study, (iv) were similar to other studies, (v) were published before January 2010, (vi) were not available in full text, (vii) did not enrol a sufficient number of subjects (<10 subjects), (viii) were not ranked on Thomson Reuters, and (ix) did not use any form of wearable sensor to measure variables associated with standing balance. Furthermore, articles were excluded if they were out-of-topic with respect to the aims of the present review, i.e., the study of standing balance using wearable sensors. Hence, we excluded studies focused on gait analysis, walking balance, fall detection, anticipatory postural adjustments, and other dynamic tasks such as sit-to-stand and Time-Up-and-Go (TUG) tests. If a study included both gait and balance analysis, we considered only the balance part of the study.

The full text of the articles that met the initial inclusion criteria were retrieved and downloaded into Mendeley Desktop 1.19.4 for further screening. To make a further selection of the large number of studies that were available for the present review, a quality assessment was performed for each included article. Full-text articles were independently assessed for suitability by two raters in terms of internal, statistical, and external validity [32] (V.A. and L.G. for papers with clinical applications and S.P. and L.G. for the remaining papers). In particular, internal validity concerns the assessment of possible biases in the research design and methods, statistical validity allows for quantifying the statistical significance of the results, and external validity is useful for assessing the generalization of the study [33]. Each rater was asked to answer a 15-item checklist similar to those commonly used in the literature for systematic and/or meta-analysis reviews [34,35,36,37,38] and modified based on the specific review topic. In particular, the proposed checklist (Table 1) provided information on (i) internal validity (question numbers 1, 3–6, and 9–11); (ii) statistical validity (question numbers 12–15); and (iii) external validity (question numbers 2–4 and 6–8). Each item of the checklist had to be answered with “Y”, “N”, or “Maybe” corresponding to scores of 1, 0, and 0.5, respectively. For each article, the total score was computed as the sum of scores of all the items in the checklist. 

Once each rater had completed the quality assessment, Cohen’s kappa statistics [39] was used to compute the degree of agreement between raters.

For each article, the final quality-assessment score was computed as the average of the scores assigned by each reviewer. The analysed articles were then divided into three different classes based on the final quality-assessment score: (i) “high quality” (final score >10), (ii) “medium quality” (final score between 5 and 10), and (iii) “low quality” (final score <5). Only articles classified as “high quality” were included in the present review.

## 3. Results

### 3.1. Searching Results and Study Selection

**Figure 1:**
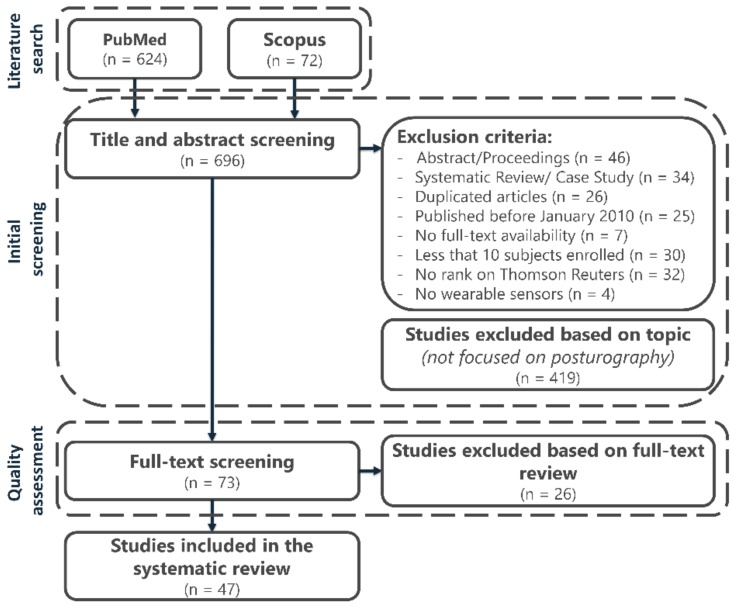
Flow diagram of the systematic search strategy and the review process.

A detailed flow diagram illustrating the searching results and the screening strategy is provided in Figure 1. A total of 696 articles was identified as eligible for inclusion in the present review. The initial screening of titles and abstracts removed 204 studies due to the previously stated exclusion criteria, which involved(ii) abstract or conference proceedings (46 articles), (iii) systematic reviews or case studies (34 articles), (iv) duplicated studies (26 articles), (v) studies published before January 2010 (25 articles), (vi) unavailable full text (7 articles), (vii) studies that enrolled less than 10 subjects (30 articles), (viii) studies not ranked on Thomson Reuters (32 articles), and (ix) studies that did not used any form of wearable sensor to measure variables related to standing balance (4 articles). A further 419 articles were removed since they were out-of-topic. The remaining 73 articles were reviewed in their full-text versions to assess their inclusion in the review after the quality check (details are in the next section). Finally, 47 high-quality articles were included in this systematic review.

### 3.2. Quality Assessment Results

Internal, statistical, and external validity were evaluated by the two raters for each of the 73 full-text papers analysed. The summary of the quality assessment is reported in Table 2. Considering the final quality-assessment score, each article was classified as low, medium, or high quality. Forty-seven articles (64.4%) were classified as high-quality contributions, 24 articles (32.9%) were classified as medium quality contributions, and 2 articles (2.7%) were classified as low-quality contributions.

The detailed results of the quality assessment performed by the raters on the 73 full-text articles are summarized in Table S1 (articles included in the systematic review) and in Table S2 (articles not included in the systematic review). The inter-rater agreement, computed by means of the Cohen’s kappa, was equal to 0.79, suggesting a good agreement between raters. After the quality assessment, 47 articles were included in this review (only those classified as “high quality”), with an average quality score of 13 ± 1 (the maximum score was 15). 

A summary of the main characteristics of the articles included is reported in Table 3.

### 3.3. Sample Population Characteristics

As detailed in Table 3, sample population characteristics and sizes varied across the included articles. The subjects enrolled in these studies consisted of healthy, young, and/or older adults (with mean age between 15 and 78 years), persons with sport-related concussions, and patients with Parkinson’s Disease (PD), Multiple Sclerosis (MS), ankle sprain, Traumatic Brain Injury (TBI), diabetic peripheral neuropathy (DPN), degenerative cerebellar ataxia, stroke, high fall risk, and haemophilia. For what concerns the patients mentioned above, a summary of the studies is provided in Table 4. The most commonly reported balance disorders were Parkinson’s disease (14 articles), degenerative cerebellar ataxia (4 articles), sport-related concussion (4 articles), and diabetic peripheral neuropathy (3 articles). 

Among the 47 studies included, 29 articles (61.7%) assessed the standing balance of pathological subjects with respect to a healthy control population, 11 articles (23.4%) assessed the standing balance only on healthy subjects, while 7 articles (14.9%) assessed the standing balance only on pathological subjects. Sample size ranged from 10 (based on the exclusion criterion) to 135 subjects.

### 3.4. Sensor Type and Placement

Several wearable sensors were used to assess standing balance. Wearable sensors included inertial motion sensors equipped with accelerometers, gyroscopes, and magnetometers; standalone multiaxial accelerometers, and smartphones equipped with inertial sensors. Of the 47 included articles, 26 articles (55.3%) used commercial inertial sensors, 13 articles (27.7%) used commercial 3D accelerometers, and the remaining 8 articles (17.0%) used one-dimensional or two-dimensional homemade accelerometers. The most commonly used inertial sensors were Opal APDM Wearable Technologies (10 articles), MTX Xsens Enschede (8 articles), and BalanSens BioSensics LLC (3 articles). A wide range of sampling frequencies (from 10 Hz to 1000 Hz) was used to acquire the signals during standing balance measurements, but the most commonly used sampling frequency was 100 Hz.

**Figure 2:**
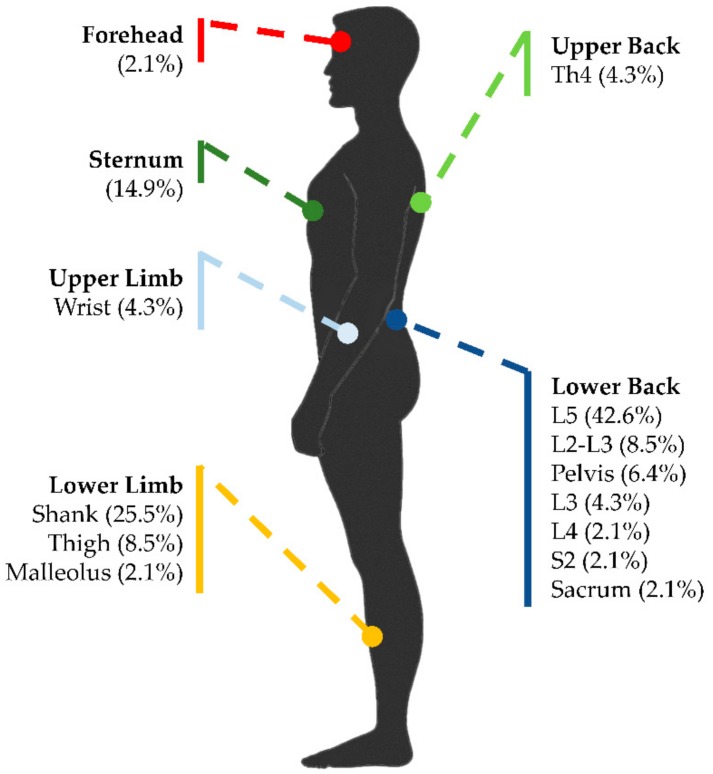
Sensor placements reported in experimental protocols with indication of the percentage of the articles included in this review that consider each position.

Similarly, several sensor placements of the wearable sensors were described in the experimental protocols, depending on the postural task. Among the 47 included articles, 38 articles (80.9%) placed the wearable sensors on the lower back near the center of mass (e.g., lumbar region of the trunk at L5 and sacral region of the trunk at S2), 15 articles (31.9%) placed it on the lower limb (e.g., thigh, malleolus, and shank), 7 articles (14.9%) placed it in correspondence with the sternum, 5 articles (10.6%) placed it on the upper back (e.g., thoracic region of the trunk at Th4), 3 articles (6.4%) placed it on the upper limb (e.g., wrists), and 1 article (2.1%) placed it on the forehead. Figure 2 represents all the sensor placements used in the reviewed articles. All the wearable sensors were attached to the subjects by means of elastic belts or Velcro^®^ bands. Further details on the type and placement of the wearable sensors used in the included articles are summarized in Table 3. 

### 3.5. Parameters for Standing-Balance Assessment

Several parameters were calculated for assessment of the standing balance from the signals acquired through the wearable sensors. The acquired signals were usually lowpass filtered by means of digital filters with cut-off frequencies that ranged between 0.5 Hz and 10 Hz. The most commonly reported parameters computed from the filtered acceleration signals were Root-Mean-Square (RMS) (21 articles) expressed in m/s^2^, jerk index (8 articles) expressed in m^2^/s^5^, range of accelerations (8 articles) expressed in m/s^2^, centroidal frequency (7 articles) expressed in Hz, and frequency dispersion (6 articles). The most frequently used parameter computed from the velocity signals (first integral of acceleration) was the mean sway velocity (12 articles) expressed in m/s. The most commonly reported parameters computed from the displacement signals (second integral of acceleration) were RMS (6 articles) expressed in mm, sway area (5 articles) expressed in mm^2^, mean distance (5 articles) expressed in mm, and sway path length (4 articles) expressed in mm.

A summary and a brief description of the balance parameters used in at least two articles is provided in Table 5, with indication of the corresponding references. Parameters used only by a single article were not reported.

#### 3.6. Validation Against a Gold Standard

Validation against a gold standard (e.g., force plate and/or clinical score) was introduced by some authors to check the sensitivity and experimental validity of the accelerometric measures (acquired through inertial sensors) compared with the standard laboratory measures (COP and clinical scores). Among the 47 articles included in the review, only 17 validated the results against a gold standard. Ten articles (21.3%) validated the results against a force plate (e.g., AMTI AccuSway-O, Kistler and Synapsis Posturography System), and the other 7 articles (14.9%) validated against a clinical score (e.g., Balance Error Scoring System (BESS) and Berg Balance Score (BBS)). Among the articles that included a validation against a gold standard, 4 articles (8.5%) also compared the test–retest reliability of wearable-sensor and force-plate measurements. A summary of the articles that included a validation against a gold standard is reported in Table 6.

## 4. Discussion

This work demonstrated that, in the literature, there is a large body of high-quality papers (47 articles) evaluating postural balance through wearable sensors. We obtained a good inter-rater agreement for the assessment of quality of the full-text papers analysed (Cohen’s kappa equal to 0.79), meaning that the raters had only minor discrepancies in their judgments of internal, statistical, and external validity concerning the articles examined.

The authors think that, in clinics, the advantages of using wearable-sensor outcome measures of balance, instead of clinical subjective scores, are evident. Wearable sensors can provide a huge amount of data that, if properly processed and correctly interpreted, may allow for assessing balance performance in a more useful, accurate, reliable, and repeatable manner. Indeed, in using wearable sensors, it is possible to easily include a large number of subjects and task repetitions, to collect data out of the lab, to engage patients in more personalized rehabilitation protocols, and to campaign to older subjects active aging and fall prevention. 

Among the many different applications, it emerged that the postural sway assessment through wearable sensors may be particularly important for Parkinson’s disease patients. This is not surprising considering the difficulties that clinicians may have in the prescription of the correct Levodopa drug dose and its fractioning and in the follow-up adjustments to therapy to control patient symptoms and the effects that the drug itself may have on balance performance [44,45,47,48,52,53,56,69,70,71,74,76,77,79].

Wearable sensor technology is widely available at low cost. In the simplest applications, the inertial sensors embedded in smartphones can be used to measure postural sway [49,65,66,69,76]. On the other hand, recently, a number of wearable systems were specifically designed to perform instrumented balance analysis. In some cases, these systems were customized for specific applications in the rehabilitation field, including systems relying on biofeedback. From a technical perspective, reviewing the articles for this work, the authors realized that there is a general lack of information pertaining to sensor calibration procedures. Commonly, two of the IMU sensing axes were oriented along the ML and AP anthropometric directions and the third axis was oriented along the vertical direction (i.e., gravity line). Considering that balance postural tasks involve quiet standing trials and small sway angles, measurements in the ML and AP directions are ideally not biased by gravity acceleration. In the reviewed paper, it was generally assumed that the components of gravity acceleration in the AP and ML directions due to sensors misalignment were negligible. Overall, little or no information is provided on this important aspect. A rigorous measurement approach requires that the estimated orientation of the sensor axes with respect to a fixed global frame is used to rotate the measured acceleration from the sensor-fixed to global frame and that the gravity constant is subtracted to obtain the net motion acceleration.

A variety of different research protocols was found in the examined articles. In many practical situations, a single sensor positioned on the lower back of the subject, mostly at the L5 level, is used to perform the posturographic examination. In some papers, additional sensors are placed on the lower limbs to assess the postural strategy (e.g., hip or ankle strategy) [44,45,59,60]. Few articles report sensor placement also on the upper limbs and trunk, but in these case, additional aims are the assessment of the base of support [61], trunk tilt [47], objective BESS [46], and correction of the vertical position of the Center of Mass (COM) [56]. In most cases, subjects are asked to maintain double leg stance for 30 s. While in the literature it is widely recognized that the position of the feet on the support surface heavily influences the postural sway, since it modifies the base of support, a standard feet position is not fully established. Indeed, the feet position sometimes is not even reported in the study protocol (n = 10 papers failed to report this information). Typical feet positions in double leg stance are: (1) feet together (opening angle: 0°, inter-malleolus distance: 0 cm); (2) feet opening angle ranging from 10° to 30° (the latter being the most frequent value), with inter-malleolus distance ranging from 0 cm up to a 10 cm; (3) self-selected feet position; and (4) footprint, having the same template position for every subject. Although one may think that the position with feet together might be easily standardized, this position can be challenging for some subjects suffering from balance-related disabilities. Patients may prefer keeping their feet apart to maintain balance. Furthermore, keeping feet apart in a comfortable self-selected position seems to provide an ecological test condition, closer to real-life upright stance. The drawback of this choice is, evidently, that the balance performance may be biased by the subjective selection of the base of support (the larger the base of support, the better the balance performance). The above-discussed issues are probably the main reasons why researchers have not yet reached a consensus on feet positioning during the examination of postural sway. In this perspective, the same debate characterized “traditional” posturography, i.e., posturography performed through force plates. However, since many current applications based on wearable sensors and many more forthcoming applications will be carried out-of-the-lab in uncontrolled environments with subjects tested at their domicile and/or during their habitual activities of daily living, the self-selected feet position might still be the best compromise.

Typically, at least two different test conditions are considered, i.e., with eyes open and closed, to estimate the effect of visual deprivation on balance. In some cases, in addition to a firm surface, a foam surface is used to differently stimulate the proprioceptive system of subjects during the postural balance task. In other cases, subjects stand in tandem, semi-tandem, or single-leg stance (on the dominant side, on the contralateral side, or alternating both conditions) in order to challenge their balance control. In a few studies, a dual task protocol is also introduced, e.g., asking subjects to count down by 3 from 100 while standing upright, to study the interference of a concomitant cognitive load on balance.

Most of the outcome measures introduced in the analysed studies are based on accelerometric signals; a few studies use gyroscope signals, and only very seldomly, signals from magnetometers are mentioned. The most frequently used outcome measure is the Root-Mean-Square (RMS) calculated from acceleration signals. This parameter is typically evaluated separately for the anteroposterior and mediolateral directions. In some cases, the total RMS is reported. With regard to acceleration signals, it should be noticed that a direct comparison with traditional force-platform (COP) signals is not possible [87]. The parameter values obtained from acceleration and COP signals estimate different physical quantities. Furthermore, wearable sensors are placed in different positions on the body (the most common location being on the back at the L5 level in correspondence to the COM) with respect to where the information from the COP signals arise (between the feet and within the base of support). In some cases, a 1-link or 2-link inverted pendulum model is applied in an attempt to bridge the gap [55,56,62,71,76,77,79,81,84]. The fact that acceleration signals obtained from wearable sensors and traditional COP signals obtained from a force platform cannot be directly compared is not a problem by itself if the concept of a new, wearable-based posturography is introduced. With this statement, the authors mean that, as long as wearable sensors provide useful information on postural balance, it is irrelevant that this information is based on parameters that are not directly comparable with those used in traditional posturography. This point of view is supported by valuable contributions such as the Instrumented test of Postural Sway (ISway) proposed by Mancini et al. in 2012 [71]. The basic idea of this kind of approach is that the new wearable technology, introducing an IMU-based assessment of the postural sway, is mature enough to “replace” balance clinical scales and scores without the limitation of the traditional posturographic approach.

Moreover, analysis of the most significative parameters associated with different balance disorders shows that, in PD populations, the parameters that best discriminate postural sway in the time domain are the jerk index [48,70,71,77], the sway amplitude [56,77], and the range of acceleration signals [76], while in the frequency domain, they are frequency dispersion [70,77] and centroidal frequency [71,79]. People with MS have increased sway acceleration amplitude [83], and instrumented standing balance measures were best for spatiotemporal measures, while frequency measures were less reliable [50]. Individuals with concussions displayed increased normalized path lengths of the acceleration signal in the AP [42] and ML [68] directions but also wider sway volume and area of the acceleration signals [54]. Among the high-quality articles selected in this review, 36% focused on the validation of wearable sensors against a gold standard approach. In particular, 10 papers were focused on the comparison between the performance of wearable sensors and force plate for postural balance assessment, while 7 papers focused on the correlations with clinical scores or scales (such as BESS or BBS). For the former, investigations were frequently limited to the evaluation of the repeatability of the wearable sensor approach compared to the traditional COP measurements, through the analysis of intra-class correlation coefficients or analogous measures. The authors noticed a lack of information on the comparison of sensitivity between wearable systems and force-plate traditional approaches. However, this is a crucial aspect. Indeed, especially in the clinical field, it is very important that the “least detectable change” of an outcome measure is smaller, enough for the specific application under consideration. Hence, the authors think that one open issue in this research field is the sensitivity of wearable systems with respect to traditional gold-standard force plates. Future studies should investigate more deeply this aspect. 

## 5. Conclusions

After a quality assessment of the selected papers, we summarized the state-of-the-art knowledge on wearable sensors used to evaluate standing balance, highlighting the main applications in clinics and active aging and discussing the best sensor location and most effective data acquisition protocols. The results of this review suggest that efforts in the validation of wearable systems against traditional posturographic approaches should focus on the evaluation of the sensitivity of the outcome measures provided by this promising technology.

## Figures and Tables

**Table 1:** Proposed checklist for the quality assessment of internal validity, statistical validity, and external validity: Reproduced and modified with permission from J. Taborri et al., Feasibility of Muscle Synergy Outcomes in Clinics, Robotics, and Sports: A Systematic Review; published by Hindawi, 2018.

Item	Index	Score		
*Aim of the work*				
1	Description of a specific, clearly stated purpose (IV)	Y	N	Maybe
2	The research question is scientifically relevant (EV)	Y	N	Maybe
	*Inclusion criteria (selection bias)*			
3	Description of inclusion and/or exclusion criteria (IV-EV)	Y	N	Maybe
*Data collection & processing (performance bias)*				
4	Data collection is clearly described and reliable (IV-EV)	Y	N	Maybe
5	Same data collection method used for all subjects (IV)	Y	N	Maybe
6	Data processing is clearly described and reliable (IV-EV)	Y	N	Maybe
*Data loss (attrition bias)*				
7	Data loss <20% (EV)	Y	N	Maybe
*Outcomes (detection bias)*				
8	Outcomes are topic relevant (EV)	Y	N	Maybe
9	Outcomes are the same for all the subjects (IV)	Y	N	Maybe
10	The work answers the scientific question stated in the aim (IV)	Y	N	Maybe
*Presentation of the results*				
11	Presentation of the results is sufficient to assess the adequacy of the analysis (IV)	Y	N	Maybe
*Statistical approach*				
12	Appropriate statistical analysis techniques (SV)	Y	N	Maybe
13	Clearly states the statistical test used (SV)	Y	N	Maybe
14	States and references the analytical software used (SV)	Y	N	Maybe
15	Sufficient number of subjects (SV)	Y	N	Maybe

**EV**: External Validity; **IV**: Internal Validity; **SV**: Statistical Validity.

**Table 2:** Summary of the quality assessment conducted by raters on the articles included in the review.

Quality	*N*	% of articles
High (score >10)	47	64.4%
Medium (score between 5 and 10)	24	32.9%
Low (score <5)	2	2.7%
**Total**	**73**	

**N**: Number of articles.

**Table 3:** Summary of the main characteristics of the articles included in the review.

First Author (Reference)	Population (Mean Age in Years ± SD)	Sensors	Sensor Placement	Test Condition(s)	Test Duration (in Each Condition)	Feet Position (Angle and Heel Distance)
Abe et al. [40]	20 persons with ankle sprain (22.7 ± 3.4) 23 controls (23.4 ± 3.5)	2 3D-accelerometers Freq: 100 Hz ACC range: ± 2 g	Lower limb (malleolus) Forehead	Single leg stance (EO), dominant side	20 s	N/A
Adamovà et al. [41]	10 degenerative cerebellar ataxia (52.2 ± 11.7) 11 controls (26.0 ± 6.4)	1 3D-inertial sensor (ACC and GYR) Freq: N/A	Lower back (L2–L3)	Double leg stance (EO/EC) Surface: firm and foam	60 s	30°, 0 cm
Alkathiry et al. [42]	56 adolescents with sport-related concussion (15 ± 1.4)	1 3D-accelerometer Freq: 50 Hz	Lower back	Double leg stance (EO/EC) Tandem (EO/EC) Surface: firm and foam	30 s	0°, 0 cm
Baracks et al. [43]	48 persons with sport-related concussion (20.6 ± 1.5) 45 controls (20.8 ± 1.4)	1 3D-inertial sensor (ACC, GYR, and MAG) Freq: N/A	Lower back (L4–L5)	Double leg stance (EC) Single leg stance (EC), nondominant side Tandem (EC)	30 s	17°, 3.8 cm
Baston et al. [44]	70 iPD (67 ± 7) 21 controls (67 ± 6)	2 3D-inertial sensors (ACC and GYR) Freq: 50 Hz	Lower back (L5) Lower limb (shank)	Double leg stance (EO)	30 s	Footprint template
Bonora et al. [45]	33 iPD-noFOG (67.5 ± 7.7) 25 iPD-FOG (67.0 ± 6.5) 13 FGD (73.3 ± 6.5) 32 controls (69.4 ± 7.1)	3 3D-inertial sensors (ACC, GYR, and MAG) Freq: 128 Hz	Lower back (L4–L5) Lower limb (shank)	Single leg stance (mini-BESS)	30 s	N/A, Shoulders
Brown et al. [46]	30 healthy (25.4 ± 4.2)	7 3D-inertial sensors (ACC and GYR) Freq: 102.4 Hz	Lower back (pelvis) Sternum Upper limb (wrist) Lower limb (shank)	BESS test Surface: firm and foam	20 s	N/A
Bzduskova et al. [47]	13 iPD (63.7 ± 5.7) 13 young controls (25.0 ± 2.3) 13 elderly controls (70.1 ± 4.5)	2 2D-accelerometers Freq: 100 Hz	Lower back (L5) Upper back (Th4)	Double leg stance (EO/EC)	20 s	Self-selected, 15 cm
Chen et al. [48]	23 iPD (66.2 ± 7.6) 23 controls (64.2 ± 7.3)	1 3D-inertial sensor (ACC, GYR, and MAG) Freq: N/A ACC range: ± 6 g	Lower back (L4–L5)	Double leg stance (EO/EC) Dual task (counting down by 3 from 1000)	30 s	Footprint template
Chiu et al. [49]	15 healthy (23.4 ± 5.3)	1 3D-accelerometer Freq: 10 Hz	Lower limb (shank)	Single leg stance (EO/EC), dominant and nondominant side	20 s	N/A
Craig et al. [50]	15 MS (48.2 ± 8.7) 15 controls (47.8 ± 9.5)	6 3D-inertial sensors (ACC, GYR, and MAG) Freq: 128 Hz	Lower back (L5) Sternum Lower limb (shank)	Double leg stance (EO)	30 s	Self-selected, 10 cm
Cruz-Montecinos et al. [51]	15 patients with haemophilia (21.8 ± 3.9) 15 controls (21.9 ± 1.4)	1 3D-accelerometer Freq: 250 Hz ACC range: ± 3 g	Lower back (L2–L3)	Double leg stance (EO/EC)	30 s	N/A
Curtze et al. [52]	104 iPD (66.5 ± 6.1)	1 3D-inertial sensors (ACC and GYR) Freq: N/A	Lower back (L5) ∙	SAW test	30 s	30°, 10 cm
De Souza Fortaleza et al. [53]	26 iPD-FOG (69.2 ± 7.9) 30 iPD-noFOG (68.6 ± 8.4)	8 3D-inertial sensors (ACC, GYR, and MAG) Freq: N/A	Lower back (L5)	SAW test Dual task (counting down by 3)	30 s	N/A
Doherty et al. [54]	15 persons with concussion (21.8 ± 3.5) 15 controls (22.5 ± 3.7)	1 3D-inertial sensor (ACC and GYR) Freq: 102.4 Hz ACC range: ± 8 g	Lower back (L3–L5)	BESS test	20 s	N/A
Ehsani et al. [55]	10 high fall risk persons (83.6 ± 9.5) 10 healthy young (23.3 ± 2.3) 10 healthy elderly (72.9 ± 2.8)	2 3D-inertial sensors (ACC and GYR) Freq: N/A	Lower limb (thigh and shank)	Double leg stance (EO/EC)	30 s	0°, 0 cm
Gago et al. [56]	10 iPD (73.0 ± N/A) 5 vPD (77 ± N/A)	1 3D-inertial sensor (ACC and GYR) Freq: 113 Hz	Lower back Lower limb (thigh and shank)	Double leg stance (EO/EC)	30 s	0°, 0 cm
Gera et al. [57]	38 mTBI (20.6 ± 1.3) 81 controls (21.0 ± 1.4)	1 3D-inertial sensor (ACC, GYR, and MAG) Freq: N/A	Lower back (L5)	Double leg stance (EO/EC) Surface: firm and foam	30 s	0°, 0 cm
Greene et al. [58]	120 healthy (73.7 ± 5.8)	1 3D-inertial sensor (ACC and GYR) Freq: 102.4 Hz	Lower back (L3)	Double leg stance (EC) Semi-tandem (EO)	40 s and 30 s	0°, 0 cm
Grewal et al. [59]	29 persons with diabetic peripheral neuropathy (57 ± 10)	2 3D-inertial sensors (ACC, GYR, and MAG) Freq: N/A	Lower back Lower limb (shank)	Double leg stance (EO/EC)	30 s	N/A, shoulders
Grewal et al. [60]	Diabetic peripheral neuropathy: - 16 in intervention group (64.9 ± 8.5) - 19 in control group (62.6 ± 7.9)	5 3D-inertial sensors (ACC, GYR, and MAG) Freq: 100 Hz	Lower back Lower limb (thigh and shank)	Double leg stance (EO) Dual task	30 s	Self-selected, self-selected
Guo et al. [61]	11 healthy (26.1 ± 4.2)	1 3D-inertial sensor (ACC and GYR) Freq: 240 Hz	Lower back (pelvis) Upper back Upper and lower limbs	Double leg stance (EO)	20 s	10°, self-selected
Halickà et al. [62]	20 healthy (22.6 ± N/A)	2 2D-accelerometers Freq: 100 Hz ACC range: ± 1.7 g	Lower back (L5) Upper back (Th4)	Double leg stance (EO) Surface: firm and foam	50 s	30°, 0 cm
Heebner et al. [63]	Healthy: - 10 in reliability group (24.3 ± 4.2) - 13 in validity group (24.1 ± 3.1)	1 3D-accelerometers Freq: 1000 Hz ACC range: ± 1.6 g	Lower back (L5)	Double leg stance (EO/EC) Single leg stance (EO/EC), dominant side Tandem (EO/EC) Surface: firm and foam	30 s	N/A
Hejda et al. [64]	10 degenerative and progressive cerebellar ataxia (52.2 ± 11.7) 11 controls (26.0 ± 6.4)	1 3D-inertial sensor (ACC and GYR) Freq: 100 Hz	Lower back (L2–L3)	Double leg stance (EO/EC) Surface: firm and foam	60 s	30°, 0 cm
Hou et al. [65]	10 chronic stroke (57.7 ± 13.3) 13 controls (45.6 ± 11.7)	1 3D-inertial sensor (ACC and GYR) Freq: 50 Hz	Lower back (S2)	Double leg stance (EO/EC) Semi-tandem (EO/EC)	30 s	Self-selected, shoulders
Hsieh et al. [66]	Elderly persons: - 22 low-risk falls (64.8 ± 4.5) - 8 high-risk falls (72.3 ± 6.6)	1 3D-accelerometer Freq: 200 Hz	Sternum	Double leg stance (EO/EC) Single leg stance (EO/EC), dominant side Tandem (EO/EC) Semi-tandem (EO/EC) Dual task (counting down by 3 from 100 or 200)	30 s	N/A
King et al. [67]	13 mTBI (16.3 ± 1.6) 13 controls (16.7 ± 2.1)	1 3D-accelerometer Freq: 120 Hz	Lower back (L5)	BESS test mBESS test iBESSm test imBESS test Surface: firm and foam	30 s	0°, 0 cm
King et al. [68]	52 persons with concussion (20.4 ± 1.3) 76 controls (20.6 ± 1.4)	1 3D-inertial sensor (ACC, GYR, and MAG) Freq: N/A	Lower back (L5)	mBESS test	30 s	N/A
Lipsmeier et al. [69]	43 iPD (57.5 ± 8.5) 35 Controls (56.2 ± 7.8)	1 3D-inertial sensor (ACC, GYR, and MAG) Freq: N/A	N/A	Double leg stance (EO)	30 s	Self-selected, self-selected
Mancini et al. [70]	13 iPD (60.4 ± 8.5) 12 controls (60.2 ± 8.2)	1 3D-inertial sensor (ACC and GYR) Freq: 100 Hz ACC range: ± 1.7 g	Lower back (L5)	Double leg stance (EO/EC)	40 s	Self-selected, 10 cm
Mancini et al. [71]	Study I: - 13 iPD (60.4 ± 8.5) - 12 controls (60.2 ± 8.2) Study II: - 17 iPD (67.1 ± 7.3) - 17 controls (67.9 ± 6.1)	1 3D-accelerometer Freq: 50 Hz ACC range: ± 1.7 g	Lower back (L5)	Double leg stance (EO)	30 s	Self-selected, 10 cm
Matheron et al. [72]	33 healthy elderly (73.4 ± 6.8)	1 3D-accelerometer Freq: 100 Hz	Lower back	Double leg stance (EO/EC) Dual task (counting down by 3 from 100)	60 s	30°, 4 cm
Melecky et al. [73]	10 persons with degenerative cerebellar ataxia (52.2 ± 11.7) 11 controls (26.0 ± 6.4)	1 3D-inertial sensor (ACC and GYR) Freq: N/A	Lower back (L2–L3)	Double leg stance (EO/EC) Surface: firm and foam	60 s	30°, 0 cm
Mellone et al. [74]	20 iPD (62 ± 7) 20 controls (64 ± 6)	1 3D-accelerometer Freq: 100 Hz ACC range: ±2 g	Lower back (L5)	Double leg stance Dual task (counting down by 3)	30 s	Footprint template
Nguyen et al. [75]	34 persons with cerebellar ataxia (47.6 ± 10.8) 22 controls (age matched)	2 3D-accelerometers Freq: 50 Hz	Upper back Sternum	Double leg stance (EO/EC)	30 s	Self-selected, 0 cm
Ozinga et al. [76]	14 iPD (63 ± 8) 14 controls (65 ± 9)	1 3D-accelerometer Freq: 100 Hz	Sacrum	Double leg stance (EO/EC)	20 s	Footprint template
Palmerini et al. [77]	20 iPD (62 ± 7) 20 controls (64 ± 6)	1 3D-accelerometer Freq: 100 Hz ACC range: ± 2 g	Lower back (L5)	Double leg stance (EO/EC) Dual task Surface: firm and foam	30 s	Footprint template
Park et al. [78]	135 healthy (57.7 ± 17.1)	6 3D-inertial sensors (ACC, GYR, and MAG) Freq: 128 Hz	Lower back (L5) Sternum	SAW test	30 s	14°, 10 cm
Rocchi et al. [79]	iPD: - 40 PIGD (64.5 ± 6.9) - 26 TD (67.6 ± 9.9) 15 controls (78.2 ± 3.9)	1 3D-accelerometer Freq: 100 Hz	Lower back	Double leg stance (EO/EC)	60 s	0°, 0 cm
Rouis et al. [80]	15 healthy (37.7 ± 15)	1 3D-accelerometer Freq: 50 Hz ACC range: ± 2 g	Lower back (L5)	Double leg stance (EO/EC)	30 s	Self-selected, self-selected
Saunders et al. [81]	20 healthy (81 ± 4)	1 3D-accelerometer Freq: 250 Hz ACC range: ± 2 g	Lower back (L3)	Double leg stance (EO/EC) Surface: firm and foam	30 s	0°, 0 cm
Solomon et al. [82]	20 MS (N/A) 20 controls (N/A)	6 3D-inertial sensors (ACC, GYR, and MAG) Freq: 120 Hz	Lower back Sternum Lower and upper limbs	SAW test Surface: foam	30 s	17.3°, 10.48 cm
Spain et al. [83]	31 MS (N/A) 28 controls (aged matched)	6 3D-inertial sensors (ACC, GYR, and MAG) Freq: 50 Hz ACC range: ± 1.7 g	Lower back (L5)	Double leg stance (EO/EC)	30 s	Footprint template
Toosizadeh et al. [84]	18 Diabetic peripheral neuropathy (65 ± 8) 18 controls (69 ± 3)	2 3D-inertial sensors (ACC, GYR, and MAG) Freq: N/A	Lower limb (thigh and shank)	Double leg stance (EO/EC)	15 s	0°, 0 cm
Whitney et al. [85]	81 healthy (47.8 ± 21.2)	1 2D-accelerometer Freq: 100 Hz ACC range: ± 1.2 g	Lower back (pelvis)	SOT test, Surface: firm and foam	40 s	N/A
Zhou et al. [86]	Diabetic peripheral neuropathy: - 78 middle-age adults (57.2 ± 4.2) - 73 older adults (71.4 ± 5.4) 45 controls (73.4 ± 6.8)	2 3D-inertial sensors (ACC and GYR) Freq: 100 Hz ACC range: ± 2 g	Lower back Lower limb (shank)	Double leg stance (EO/EC) Semi-tandem (EO)	30 s	0°, 0 cm

**ACC**: accelerometer; **BESS**: Balance Error Scoring System; **EC**: eyes closed condition; **EO**: eyes open condition; **FGD**: Frontal Gait Disorder; **FOG**: Freezing of Gait; **GYR**: gyroscope; **iPD**: idiopathic Parkinson’s Disease; **MAG**: magnetometer; **mTBI**: mild Traumatic Brain Injury; **N/A**: Not Available; **MS**: Multiple Sclerosis; **PIGD**: Postural Instability Gait Difficulty; **SAW**: Stand and Walk test; **SOT**: Sensory Organization Test; **TD**: Tremor Dominant; **vPD**: vascular Parkinson’s Disease.

**Table 4:** Summary of the balance disorders reported in the included articles.

Balance Disorder	*N*	% of Articles	Reference(s)
Parkinson’s Disease (PD)	14	29.8%	[44,45,47,48,52,53,56,69,70,71,74,76,77,79]
Degenerative Cerebellar Ataxia	4	8.5%	[41,64,73,75]
Concussion	4	8.5%	[42,43,54,68]
Diabetic Peripheral Neuropathy (DPN)	4	8.5%	[55,59,75,86]
Multiple Sclerosis (MS)	3	6.4%	[50,82,83]
High fall risk	2	4.3%	[55,66]
Traumatic Brain Injury (TBI)	2	4.3%	[57,67]
Ankle sprain	1	2.1%	[40]
Stroke	1	2.1%	[65]
Haemophilia	1	2.1%	[51]
**Total**	**36**	**76.6%**	

**N**: Number of articles.

**Table 5:** Summary and brief description of the principal balance parameters.

Balance Measure (Acceleration)	Domain	Definition of Measure	References
Range	Time	Range of acceleration signals in AP and/or ML directions (m/s^2^)	[50,71,74,76,78,80,82,85]
Root Mean Square (RMS)	Time	RMS of the accelerations in AP and/or ML directions (m/s^2^)	[40,43,44,48,50,51,52,53,58,63,66,71,74,75,78,80,81,82,83,85]
Mean Acceleration	Time	Average of the AP and/or ML accelerations (m/s^2^)	[49,80]
Mean Distance	Time	Mean distance from the center of acceleration trajectory normalized with respect to the duration of the measurement (m/s^2^)	[50,71,78]
Sway Path Length (SPL)	Time	Total accelerometer trajectory length (m/s^2^)	[41,42,50,71,76,80,82,85]
Sway Area (SA)	Time	Area spanned from the acceleration signals normalized with respect to the duration of the measurement (mm^2^/s^5^)	[50,57,71,78,80]
95% Ellipse Sway Area	Time	Elliptical area that encapsulates the sway path derived from the AP and ML accelerations (m^2^/s^4^)	[43,76,82]
95% Ellipse Sway Normalized Area	Time	Elliptical area that encapsulates the sway path derived from the AP and ML accelerations normalized with respect to the duration of the measurement (m^2^/s^5^)	[71,78]
Jerk Index (JI)	Time	Function of the time derivative of the acceleration: it is an index of sway smoothness (m^2^/s^5^).	[48,50,53,71,74,77,78,82]
Normalized Jerk Index (nJI)	Time	Jerk index normalized to range of acceleration excursion and duration (dimensionless)	[52,77,78,83]
F50	Frequency	Frequency containing 50% of the total power (Hz)	[71,77,80]
F95	Frequency	Frequency containing 95% of the total power (Hz)	[50,70,71,74,77,80]
Total Power	Frequency	Total power of the spectrum of accelerations (m^2^/s^4^)	[68,71,80,82]
Frequency Dispersion (FD)	Frequency	Measure of the variability of the frequency content of the power spectral density (0 for a pure sinusoid: it increases with spectral bandwidth to 1) (dimensionless)	[50,52,70,71,77,78]
Centroidal Frequency (CF)	Frequency	Frequency at which spectral mass is concentrated: the power of the acceleration signals above and below CF are exactly balanced (Hz).	[52,71,74,77,78,79,83]
Mean Frequency	Frequency	Mean frequency of the acceleration power spectrum (Hz)	[50,58,71]
Entropy	Frequency	Power spectrum entropy of accelerations (dimensionless)	[58,75,77]
Mean Sway Velocity (MV)	Time	First integral of the acceleration signals in AP and/or ML directions (m/s)	[52,58,69,70,71,74,77,78,79,80,82,83]
Root Mean Square (RMS)	Time	RMS of the displacements in AP and/or ML directions (mm).	[44,51,58,62,72,77]
Mean Distance (MD)	Time	Mean distance from the center of COM (mm)	[56,58,77,79]
Range	Time	Range of COM displacement (mm)	[56,77,84]
Sway Path Length (SPL)	Time	Total COM trajectory length (mm)	[56,58,77,79]
Sway Area (SA)	Time	Area included in the COM displacement (mm^2^ or cm^2^)	[59,60,77,84,86]
95% Ellipse Sway Normalized Area	Time	Elliptical area that encapsulates the sway path derived from the AP and ML displacement normalized with respect to the duration of the measurement (mm^2^/s)	[58,72]

**AP**: Anteroposterior direction; **COM**: Center of Mass; **ML**: Mediolateral direction.

**Table 6:** Articles with validation against a gold standard (force plate or clinical score).

Validation	*N*	% of Articles	References
Force plate	10	21.3%	[54,62,63,64,66,70,71,73,80,85]
Clinical score	7	14.9%	[46,54,58,61,65,67,68]
**Total**	**17**	**36.2%**	

**N**: Number of articles.

## Supplementary Materials

The following are available online at https://www.mdpi.com/1424-8220/19/19/4075/s1, Table S1: Results of the quality assessment performed by the raters on the 47 full-text articles. Table S2: Results of the quality assessment performed by raters on the articles not included in the systematic review.

## Abbreviations

The following abbreviations are used in the manuscript:

ACC	Accelerometer
AP	Anteroposterior
BBS	Berg Balance Scale
BESS	Balance Error Scoring System
CF	Centroidal frequency
COM	Center of Mass
COP	Center of Pression
DPN	Diabetic Peripheral Neuropathy
EO	Eyes Open
EC	Eyes Closed
EV	External Validity
FD	Frequency Dispersion
FGD	Frontal Gait Disorder
FOG	Freezing of Gait
GYR	Gyroscope
IMU	Inertial Measurement Unit
iPD	Idiopathic Parkinson’s Disease
IV	Internal Validity
JI	Jerk Index
MAG	Magnetometer
MD	Mean Distance
MIMU	Magneto Inertial Measurement Unit
ML	Mediolateral
MS	Multiple Sclerosis
MV	Mean Sway Velocity
N/A	Not Available
nJI	Normalized Jerk Index
PD	Parkinson’s Disease
PIGD	Postural Instability Gait Difficulty
RMS	Root Mean Square
SA	Sway Area
SAW	Stand and Walk
SOT	Sensory Organization Test
SPL	Sway Path Length
TBI	Traumatic Brain Injury
TD	Tremor Dominant
vPD	Time-Up-and-GO Test
TUG	Vascular Parkinson’s Disease

## References

[1] Piirtola M., Era P. (2006). Force platform measurements as predictors of falls among older people—A review. Gerontology.

[2] Fioretti S., Guidi M., Ladislao L., Ghetti G. Analysis and reliability of posturographic parameters in Parkinson patients at an early stage. Proceedings of the 26th Annual International Conference of the IEEE Engineering in Medicine and Biology Society.

[3] Agostini V., Chiaramello E., Bredariol C., Cavallini C., Knaflitz M. (2011). Postural control after traumatic brain injury in patients with neuro-ophthalmic deficits. Gait Posture.

[4] Maranesi E., Ghetti G., Rabini R.A., Fioretti S. (2014). Functional reach test: Movement strategies in diabetic subjects. Gait Posture.

[5] Agostini V., Sbrollini A., Cavallini C., Busso A., Pignata G., Knaflitz M. (2016). The role of central vision in posture: Postural sway adaptations in Stargardt patients. Gait Posture.

[6] Agostini V., Chiaramello E., Canavese L., Bredariol C., Knaflitz M. (2013). Postural sway in volleyball players. Hum. Mov. Sci..

[7] Chaudhry H., Bukiet B., Ji Z., Findley T. (2011). Measurement of balance in computer posturography: Comparison of methods—A brief review. J. Bodyw. Mov. Ther..

[8] Neville C., Ludlow C., Rieger B. (2015). Measuring postural stability with an inertial sensor: Validity and sensitivity. Med. Devices Evid. Res..

[9] Weiss A., Herman T., Plotnik M., Brozgol M., Maidan I., Giladi N., Gurevich T., Hausdorff J.M. (2010). Can an accelerometer enhance the utility of the Timed Up & Go Test when evaluating patients with Parkinson’s disease?. Med. Eng. Phys..

[10] Kim J.H., Sienko K.H. (2009). The Design of a Cell-Phone Based Balance-Training Device. J. Med. Devices.

[11] Giggins O.M., Sweeney K.T., Caulfield B. (2014). Rehabilitation exercise assessment using inertial sensors: A cross-sectional analytical study. J. Neuroeng. Rehabilit..

[12] Leardini A., Lullini G., Giannini S., Berti L., Ortolani M., Caravaggi P. (2014). Validation of the angular measurements of a new inertial-measurement-unit based rehabilitation system: Comparison with state-of-the-art gait analysis. J. Neuroeng. Rehabilit..

[13] Grimm B., Bolink S. (2016). Evaluating physical function and activity in the elderly patient using wearable motion sensors. EFORT Open Rev..

[14] Horak F., King L., Mancini M. (2015). Role of Body-Worn Movement Monitor Technology for Balance and Gait Rehabilitation. Phys. Ther..

[15] Mileti I., Taborri J., Rossi S., Prete Z.D., Paoloni M., Suppa A., Palermo E. Measuring age-related differences in kinematic postural strategies under yaw perturbation. Proceedings of the 2018 IEEE International Symposium on Medical Measurements and Applications (MeMeA).

[16] Mancini M., Horak F.B. (2010). The relevance of clinical balance assessment tools to differentiate balance deficits. Eur. J. Phys. Rehabilit. Med..

[17] Özdemir A.T., Barshan B. (2014). Detecting falls with wearable sensors using machine learning techniques. Sensors.

[18] Shany T., Redmond S.J., Narayanan M.R., Lovell N.H. (2012). Sensors-based wearable systems for monitoring of human movement and falls. IEEE Sens. J..

[19] Howcroft J., Kofman J., Lemaire E.D. (2013). Review of fall risk assessment in geriatric populations using inertial sensors. J. Neuroeng. Rehabilit..

[20] Roeing K.L., Hsieh K.L., Sosnoff J.J. (2017). A systematic review of balance and fall risk assessments with mobile phone technology. Arch. Gerontol. Geriatr..

[21] Sun R., Sosnoff J.J. (2018). Novel sensing technology in fall risk assessment in older adults: A systematic review. BMC Geriatr..

[22] Pang I., Okubo Y., Sturnieks D., Lord S.R., Brodie M.A. (2019). Detection of Near Falls Using Wearable Devices: A Systematic Review. J. Geriatr. Phys. Ther..

[23] Tedesco S., Barton J., O’Flynn B. (2017). A Review of Activity Trackers for Senior Citizens: Research Perspectives, Commercial Landscape and the Role of the Insurance Industry. Sensors.

[24] Maetzler W., Domingos J., Srulijes K., Ferreira J.J., Bloem B.R. (2013). Quantitative wearable sensors for objective assessment of Parkinson’s disease. Mov. Disord..

[25] Hubble R.P., Naughton G.A., Silburn P.A., Cole M.H. (2015). Wearable Sensor Use for Assessing Standing Balance and Walking Stability in People with Parkinson’s Disease: A Systematic Review. PLoS ONE.

[26] Godinho C., Domingos J., Cunha G., Santos A.T., Fernandes R.M., Abreu D., Goncalves N., Matthews H., Isaacs T., Duffen J. (2016). A systematic review of the characteristics and validity of monitoring technologies to assess Parkinson’s disease. J. Neuroeng. Rehabilit..

[27] Sun R., McGinnis R., Sosnoff J.J. (2018). Novel technology for mobility and balance tracking in patients with multiple sclerosis: A systematic review. Expert Rev. Neurother..

[28] Ma C.Z.-H., Wong D.W.-C., Lam W.K., Wan A.H.-P., Lee W.C.-C. (2016). Balance Improvement Effects of Biofeedback Systems with State-of-the-Art Wearable Sensors: A Systematic Review. Sensors.

[29] Gordt K., Gerhardy T., Najafi B., Schwenk M. (2018). Effects of Wearable Sensor-Based Balance and Gait Training on Balance, Gait, and Functional Performance in Healthy and Patient Populations: A Systematic Review and Meta-Analysis of Randomized Controlled Trials. Gerontology.

[30] Moral-Munoz J.A., Esteban-Moreno B., Herrera-Viedma E., Cobo M.J., Perez I.J. (2018). Smartphone Applications to Perform Body Balance Assessment: A Standardized Review. J. Med. Syst..

[31] Moher D., Liberati A., Tetzlaff J., Altman D.G. (2009). Preferred reporting items for systematic reviews and meta-analyses: The PRISMA statement. J. Clin. Epidemiol..

[32] Cooper H.M., Publication S. (2015). Research Synthesis and Meta-Analysis: A Step-by-Step Approach.

[33] Slack M.K., Draugalis J.R. (2001). Establishing the internal and external validity of experimental studies. Am. J. Heal. Pharm..

[34] Kuijpers T., van der Windt D.A.W.M., van der Heijden G.J.M.G., Bouter L.M. (2004). Systematic review of prognostic cohort studies on shoulder disorders. Pain.

[35] Luppino F.S., de Wit L.M., Bouvy P.F., Stijnen T., Cuijpers P., Penninx B.W.J.H., Zitman F.G. (2010). Overweight, obesity, and depression: A systematic review and meta-analysis of longitudinal studies. Arch. Gen. Psychiatry.

[36] Alexiou K.I., Roushias A., Varitimidis S.E., Malizos K.N. (2018). Quality of life and psychological consequences in elderly patients after a hip fracture: A review. Clin. Interv. Aging.

[37] Van der Kooy K., van Hout H., Marwijk H., Marten H., Stehouwer C., Beekman A. (2007). Depression and the risk for cardiovascular diseases: Systematic review and meta analysis. Int. J. Geriatr. Psychiatry.

[38] Hagströmer M., Ainsworth B.E., Kwak L., Bowles H.R. (2012). A checklist for evaluating the methodological quality of validation studies on self-report instruments for physical activity and sedentary behavior. J. Phys. Act. Health.

[39] Cohen J. (1960). A coefficient of agreement for nominal scales. Educ. Psychol. Meas..

[40] Abe Y., Sugaya T., Sakamoto M. (2014). Postural Control Characteristics during Single Leg Standing of Individuals with a History of Ankle Sprain: Measurements Obtained Using a Gravicorder and Head and Foot Accelerometry. J. Phys. Ther. Sci..

[41] Adamová B., Kutilek P., Cakrt O., Svoboda Z., Viteckova S., Smrcka P. (2018). Quantifying postural stability of patients with cerebellar disorder during quiet stance using three-axis accelerometer. Biomed. Signal Process. Control.

[42] Alkathiry A.A., Sparto P.J., Freund B., Whitney S.L., Mucha A., Furman J.M., Collins M.W., Kontos A.P. (2018). Using Accelerometers to Record Postural Sway in Adolescents With Concussion: A Cross-Sectional Study. J. Athl. Train..

[43] Baracks J., Casa D.J., Covassin T., Sacko R., Scarneo S.E., Schnyer D., Yeargin S.W., Neville C. (2018). Acute Sport-Related Concussion Screening for Collegiate Athletes Using an Instrumented Balance Assessment. J. Athl. Train..

[44] Baston C., Mancini M., Rocchi L., Horak F. (2016). Effects of Levodopa on Postural Strategies in Parkinson’s disease. Gait Posture.

[45] Bonora G., Mancini M., Carpinella I., Chiari L., Ferrarin M., Nutt J.G., Horak F.B. (2017). Investigation of Anticipatory Postural Adjustments during One-Leg Stance Using Inertial Sensors: Evidence from Subjects with Parkinsonism. Front. Neurol..

[46] Brown H.J., Siegmund G.P., Guskiewicz K.M., van den doel K., Cretu E., Blouin J.-S. (2014). Development and Validation of an Objective Balance Error Scoring System. Med. Sci. Sports Exerc..

[47] Bzduskova D., Valkovic P., Hirjakova Z., Kimijanova J., Hlavacka F. (2018). Parkinson’s disease versus ageing: Different postural responses to soleus muscle vibration. Gait Posture.

[48] Chen T., Fan Y., Zhuang X., Feng D., Chen Y., Chan P., Du Y. (2018). Postural sway in patients with early Parkinson’s disease performing cognitive tasks while standing. Neurol. Res..

[49] Chiu Y.-L., Tsai Y.-J., Lin C.-H., Hou Y.-R., Sung W.-H. (2017). Evaluation of a smartphone-based assessment system in subjects with chronic ankle instability. Comput. Methods Programs Biomed..

[50] Craig J.J., Bruetsch A.P., Lynch S.G., Horak F.B., Huisinga J.M. (2017). Instrumented balance and walking assessments in persons with multiple sclerosis show strong test-retest reliability. J. Neuroeng. Rehabilit..

[51] Cruz-Montecinos C., De la Fuente C., Rivera-Lillo G., Morales-Castillo S., Soto-Arellano V., Querol F., Pérez-Alenda S. (2017). Sensory strategies of postural sway during quiet stance in patients with haemophilic arthropathy. Haemophilia.

[52] Curtze C., Nutt J.G., Carlson-Kuhta P., Mancini M., Horak F.B. (2016). Objective Gait and Balance Impairments Relate to Balance Confidence and Perceived Mobility in People With Parkinson Disease. Phys. Ther..

[53] De Souza Fortaleza A.C., Mancini M., Carlson-Kuhta P., King L.A., Nutt J.G., Chagas E.F., Freitas I.F., Horak F.B. (2017). Dual task interference on postural sway, postural transitions and gait in people with Parkinson’s disease and freezing of gait. Gait Posture.

[54] Doherty C., Zhao L., Ryan J., Komaba Y., Inomata A., Caulfield B. (2017). Quantification of postural control deficits in patients with recent concussion: An inertial-sensor based approach. Clin. Biomech..

[55] Ehsani H., Mohler J., Marlinski V., Rashedi E., Toosizadeh N. (2018). The influence of mechanical vibration on local and central balance control. J. Biomech..

[56] Gago M.F., Fernandes V., Ferreira J., Silva H., Rodrigues M.L., Rocha L., Bicho E., Sousa N. (2015). The effect of levodopa on postural stability evaluated by wearable inertial measurement units for idiopathic and vascular Parkinson’s disease. Gait Posture.

[57] Gera G., Chesnutt J., Mancini M., Horak F.B., King L.A. (2018). Inertial Sensor-Based Assessment of Central Sensory Integration for Balance after Mild Traumatic Brain Injury. Proc. Mil. Med..

[58] Greene B.R., McGrath D., Walsh L., Doheny E.P., McKeown D., Garattini C., Cunningham C., Crosby L., Caulfield B., Kenny R.A. (2012). Quantitative falls risk estimation through multi-sensor assessment of standing balance. Physiol. Meas..

[59] Grewal G.S., Sayeed R., Schwenk M., Bharara M., Menzies R., Talal T.K., Armstrong D.G., Najafi B. (2013). Balance rehabilitation: Promoting the role of virtual reality in patients with diabetic peripheral neuropathy. J. Am. Podiatr. Med. Assoc..

[60] Grewal G.S., Schwenk M., Lee-Eng J., Parvaneh S., Bharara M., Menzies R.A., Talal T.K., Armstrong D.G., Najafi B. (2015). Sensor-Based Interactive Balance Training with Visual Joint Movement Feedback for Improving Postural Stability in Diabetics with Peripheral Neuropathy: A Randomized Controlled Trial. Gerontology.

[61] Guo L., Xiong S. (2017). Accuracy of Base of Support Using an Inertial Sensor Based Motion Capture System. Sensors.

[62] Halická Z., Lobotková J., Bučková K., Hlavačka F. (2014). Effectiveness of different visual biofeedback signals for human balance improvement. Gait Posture.

[63] Heebner N.R., Akins J.S., Lephart S.M., Sell T.C. (2015). Reliability and validity of an accelerometry based measure of static and dynamic postural stability in healthy and active individuals. Gait Posture.

[64] Hejda J., Cakrt O., Socha V., Schlenker J., Kutilek P. (2015). 3-D trajectory of body sway angles: A technique for quantifying postural stability. Biocybern. Biomed. Eng..

[65] Hou Y.-R., Chiu Y.-L., Chiang S.-L., Chen H.-Y., Sung W.-H. (2018). Feasibility of a smartphone-based balance assessment system for subjects with chronic stroke. Comput. Methods Programs Biomed..

[66] Hsieh K.L., Roach K.L., Wajda D.A., Sosnoff J.J. (2019). Smartphone technology can measure postural stability and discriminate fall risk in older adults. Gait Posture.

[67] King L.A., Horak F.B., Mancini M., Pierce D., Priest K.C., Chesnutt J., Sullivan P., Chapman J.C. (2014). Instrumenting the Balance Error Scoring System for Use With Patients Reporting Persistent Balance Problems After Mild Traumatic Brain Injury. Arch. Phys. Med. Rehabilit..

[68] King L.A., Mancini M., Fino P.C., Chesnutt J., Swanson C.W., Markwardt S., Chapman J.C. (2017). Sensor-Based Balance Measures Outperform Modified Balance Error Scoring System in Identifying Acute Concussion. Ann. Biomed. Eng..

[69] Lipsmeier F., Taylor K.I., Kilchenmann T., Wolf D., Scotland A., Schjodt-Eriksen J., Cheng W.-Y., Fernandez-Garcia I., Siebourg-Polster J., Jin L. (2018). Evaluation of smartphone-based testing to generate exploratory outcome measures in a phase 1 Parkinson’s disease clinical trial. Mov. Disord..

[70] Mancini M., Horak F.B., Zampieri C., Carlson-Kuhta P., Nutt J.G., Chiari L. (2011). Trunk accelerometry reveals postural instability in untreated Parkinson’s disease. Parkinsonism Relat. Disord..

[71] Mancini M., Salarian A., Carlson-Kuhta P., Zampieri C., King L., Chiari L., Horak F.B. (2012). ISway: A sensitive, valid and reliable measure of postural control. J. Neuroeng. Rehabilit..

[72] Matheron E., Yang Q., Delpit-Baraut V., Dailly O., Kapoula Z. (2016). Active ocular vergence improves postural control in elderly as close viewing distance with or without a single cognitive task. Neurosci. Lett..

[73] Melecky R., Socha V., Kutilek P., Hanakova L., Takac P., Schlenker J., Svoboda Z. (2016). Quantification of Trunk Postural Stability Using Convex Polyhedron of the Time-Series Accelerometer Data. J. Healthc. Eng..

[74] Mellone S., Palmerini L., Cappello A., Chiari L. (2011). Hilbert-Huang-based tremor removal to assess postural properties from accelerometers. IEEE Trans. Biomed. Eng..

[75] Nguyen N., Phan D., Pathirana P.N., Horne M., Power L., Szmulewicz D. (2018). Quantification of Axial Abnormality Due to Cerebellar Ataxia with Inertial Measurements. Sensors.

[76] Ozinga S.J., Linder S.M., Alberts J.L. (2017). Use of Mobile Device Accelerometry to Enhance Evaluation of Postural Instability in Parkinson Disease. Arch. Phys. Med. Rehabilit..

[77] Palmerini L., Rocchi L., Mellone S., Valzania F., Chiari L. (2011). Feature selection for accelerometer-based posture analysis in Parkinson’s disease. IEEE Trans. Inf. Technol. Biomed..

[78] Park J.-H., Mancini M., Carlson-Kuhta P., Nutt J.G., Horak F.B. (2016). Quantifying effects of age on balance and gait with inertial sensors in community-dwelling healthy adults. Exp. Gerontol..

[79] Rocchi L., Palmerini L., Weiss A., Herman T., Hausdorff J.M. (2014). Balance testing with inertial sensors in patients with Parkinson’s disease: Assessment of motor subtypes. IEEE Trans. Neural Syst. Rehabilit. Eng..

[80] Rouis A., Rezzoug N., Gorce P. (2014). Validity of a low-cost wearable device for body sway parameter evaluation. Comput. Methods Biomech. Biomed. Engin..

[81] Saunders N.W., Koutakis P., Kloos A.D., Kegelmeyer D.A., Dicke J.D., Devor S.T. (2015). Reliability and validity of a wireless accelerometer for the assessment of postural sway. J. Appl. Biomech..

[82] Solomon A.J., Jacobs J.V., Lomond K.V., Henry S.M. (2015). Detection of postural sway abnormalities by wireless inertial sensors in minimally disabled patients with multiple sclerosis: A case–control study. J. Neuroeng. Rehabilit..

[83] Spain R., George R.S., Salarian A., Mancini M., Wagner J.M., Horak F.B., Bourdette D. (2012). Body-worn motion sensors detect balance and gait deficits in people with multiple sclerosis who have normal walking speed. Gait Posture.

[84] Toosizadeh N., Mohler J., Armstrong D.G., Talal T.K., Najafi B. (2015). The influence of diabetic peripheral neuropathy on local postural muscle and central sensory feedback balance control. PLoS ONE.

[85] Whitney S., Roche J., Marchetti G., Lin C., Steed D., Furman G., Musolino M., Redfern M. (2011). A comparison of accelerometry and center of pressure measures during computerized dynamic posturography: A measure of balance. Gait Posture.

[86] Zhou H., Al-Ali F., Rahemi H., Kulkarni N., Hamad A., Ibrahim R., Talal T.K., Najafi B. (2018). Hemodialysis Impact on Motor Function beyond Aging and Diabetes-Objectively Assessing Gait and Balance by Wearable Technology. Sensors.

[87] Agostini V., Aiello E., Fortunato D., Gastaldi L., Knaflitz M., Torino P. A Wearable Device to Assess Postural Sway. Proceedings of the 2019 IEEE 23rd International Symposium on Consumer Technologies (ISCT).

