# Supplementary material for: Wearable Inertial Sensors to Assess Standing Balance: A Systematic Review
Source: Sensors (Basel). 2019 Sep 20;19(19):4075. doi: 10.3390/s19194075 (PMC6806601; doi:10.3390/s19194075)
Supplement: Supplementary file 1 [file sensors-19-04075-s001.zip › Supplementary_S1.docx]

**Table S1.** Results of the quality assessment conducted by raters on the articles included in the review.

| **First Author (Reference)** | **1** | | **2** | | **3** | | **4** | | **5** | | **6** | | **7** | | **8** | | **9** | | **10** | | **11** | | **12** | | **13** | | **14** | | **15** | |
| --- | --- | --- | --- | --- | --- | --- | --- | --- | --- | --- | --- | --- | --- | --- | --- | --- | --- | --- | --- | --- | --- | --- | --- | --- | --- | --- | --- | --- | --- | --- |
|  | **R1** | **R2** | **R1** | **R2** | **R1** | **R2** | **R1** | **R2** | **R1** | **R2** | **R1** | **R2** | **R1** | **R2** | **R1** | **R2** | **R1** | **R2** | **R1** | **R2** | **R1** | **R2** | **R1** | **R2** | **R1** | **R2** | **R1** | **R2** | **R1** | **R2** |
| Abe et al. [40] | 0.5 | 0.5 | 0 | 0.5 | 1 | 1 | 0.5 | 0 | 1 | 1 | 0 | 0.5 | 1 | 1 | 1 | 0.5 | 1 | 1 | 1 | 1 | 1 | 1 | 1 | 1 | 1 | 1 | 1 | 1 | 1 | 1 |
| Adamovà et al. [41] | 0.5 | 1 | 1 | 1 | 1 | 1 | 0.5 | 1 | 1 | 1 | 1 | 1 | 1 | 1 | 1 | 1 | 1 | 1 | 0.5 | 1 | 1 | 0.5 | 1 | 0 | 1 | 1 | 1 | 1 | 0.5 | 0 |
| Alkathiry et al. [42] | 1 | 1 | 1 | 0.5 | 1 | 1 | 1 | 1 | 1 | 1 | 1 | 1 | 1 | 1 | 1 | 0.5 | 1 | 1 | 0.5 | 0.5 | 1 | 1 | 1 | 1 | 1 | 1 | 1 | 1 | 1 | 1 |
| Baracks et al. [43] | 1 | 1 | 1 | 0.5 | 1 | 0.5 | 1 | 1 | 1 | 1 | 1 | 1 | 1 | 1 | 1 | 1 | 1 | 1 | 1 | 1 | 1 | 1 | 1 | 1 | 1 | 1 | 1 | 1 | 1 | 1 |
| Baston et al. [44] | 1 | 1 | 1 | 1 | 1 | 1 | 1 | 1 | 1 | 1 | 1 | 1 | 1 | 1 | 1 | 1 | 1 | 1 | 1 | 1 | 1 | 1 | 1 | 1 | 1 | 1 | 1 | 1 | 1 | 1 |
| Bonora et al. [45] | 1 | 1 | 1 | 1 | 1 | 1 | 0.5 | 1 | 1 | 1 | 1 | 1 | 0 | 0 | 1 | 1 | 1 | 0.5 | 1 | 1 | 1 | 1 | 1 | 1 | 1 | 1 | 1 | 1 | 1 | 1 |
| Brown et al. [46] | 1 | 1 | 1 | 1 | 1 | 1 | 1 | 1 | 1 | 1 | 0.5 | 0.5 | 1 | 1 | 1 | 0.5 | 1 | 1 | 1 | 0.5 | 1 | 1 | 1 | 1 | 1 | 1 | 1 | 1 | 1 | 1 |
| Bzduskova et al. [47] | 0.5 | 1 | 0 | 0 | 1 | 1 | 1 | 1 | 1 | 1 | 1 | 1 | 1 | 1 | 1 | 0.5 | 1 | 1 | 1 | 1 | 1 | 1 | 0.5 | 0.5 | 1 | 1 | 1 | 1 | 0 | 0 |
| Chen et al. [48] | 1 | 1 | 1 | 1 | 1 | 1 | 1 | 1 | 1 | 1 | 0.5 | 0.5 | 1 | 1 | 1 | 1 | 1 | 1 | 0.5 | 1 | 1 | 0.5 | 0.5 | 1 | 1 | 1 | 1 | 1 | 0.5 | 1 |
| Chiu et al. [49] | 1 | 1 | 1 | 0.5 | 0 | 0.5 | 0 | 0.5 | 1 | 1 | 0 | 0 | 1 | 1 | 1 | 1 | 1 | 1 | 1 | 1 | 1 | 1 | 0 | 0.5 | 1 | 1 | 1 | 1 | 1 | 0.5 |
| Craig et al. [50] | 1 | 1 | 1 | 1 | 1 | 1 | 1 | 1 | 1 | 1 | 1 | 0.5 | 1 | 1 | 1 | 1 | 1 | 1 | 1 | 1 | 0 | 0 | 1 | 1 | 1 | 1 | 1 | 1 | 0.5 | 0.5 |
| Cruz-Montecinos et al. [51] | 1 | 1 | 1 | 0.5 | 1 | 1 | 1 | 1 | 1 | 1 | 0.5 | 0.5 | 1 | 1 | 1 | 1 | 1 | 1 | 1 | 1 | 0.5 | 1 | 1 | 1 | 1 | 1 | 1 | 1 | 0.5 | 0 |
| Curtze et al. [52] | 1 | 1 | 1 | 1 | 1 | 1 | 1 | 0.5 | 1 | 1 | 1 | 0.5 | 1 | 1 | 1 | 0.5 | 1 | 1 | 0.5 | 1 | 1 | 0.5 | 0.5 | 0.5 | 1 | 1 | 0 | 0 | 1 | 1 |
| De Souza Fortaleza et al. [53] | 1 | 1 | 0.5 | 1 | 1 | 1 | 1 | 1 | 1 | 1 | 1 | 1 | 1 | 1 | 1 | 1 | 1 | 1 | 1 | 1 | 1 | 0 | 1 | 1 | 1 | 1 | 1 | 1 | 1 | 1 |

**Table S1.** Results of the quality assessment conducted by raters on the articles included in the review. (*Continued*)

| **First Author (Reference)** | **1** | | **2** | | **3** | | **4** | | **5** | | **6** | | **7** | | **8** | | **9** | | **10** | | **11** | | **12** | | **13** | | **14** | | **15** | |
| --- | --- | --- | --- | --- | --- | --- | --- | --- | --- | --- | --- | --- | --- | --- | --- | --- | --- | --- | --- | --- | --- | --- | --- | --- | --- | --- | --- | --- | --- | --- |
|  | **R1** | **R2** | **R1** | **R2** | **R1** | **R2** | **R1** | **R2** | **R1** | **R2** | **R1** | **R2** | **R1** | **R2** | **R1** | **R2** | **R1** | **R2** | **R1** | **R2** | **R1** | **R2** | **R1** | **R2** | **R1** | **R2** | **R1** | **R2** | **R1** | **R2** |
| Doherty et al. [54] | 1 | 1 | 1 | 1 | 1 | 1 | 0.5 | 1 | 1 | 1 | 0 | 0.5 | 1 | 1 | 1 | 1 | 1 | 1 | 1 | 1 | 0 | 0 | 0.5 | 0.5 | 1 | 1 | 1 | 1 | 1 | 0.5 |
| Ehsani et al. [55] | 0.5 | 0.5 | 0.5 | 0.5 | 1 | 1 | 0 | 0.5 | 1 | 1 | 0.5 | 0.5 | 1 | 1 | 1 | 0.5 | 1 | 1 | 1 | 1 | 1 | 1 | 1 | 1 | 1 | 1 | 1 | 1 | 0 | 0 |
| Gago et al. [56] | 1 | 1 | 1 | 0.5 | 1 | 1 | 0.5 | 1 | 1 | 1 | 0.5 | 1 | 1 | 1 | 1 | 1 | 1 | 0.5 | 0.5 | 1 | 1 | 1 | 1 | 1 | 1 | 1 | 1 | 1 | 0 | 0 |
| Gera et al. [57] | 1 | 1 | 1 | 1 | 1 | 1 | 1 | 1 | 1 | 1 | 0 | 0.5 | 1 | 1 | 1 | 1 | 1 | 1 | 1 | 1 | 1 | 1 | 1 | 0.5 | 1 | 1 | 0 | 0 | 1 | 1 |
| Greene et al.  [58] | 1 | 1 | 1 | 1 | 1 | 1 | 0.5 | 1 | 0.5 | 1 | 0.5 | 0.5 | 1 | 1 | 1 | 1 | 1 | 1 | 1 | 1 | 0 | 0.5 | 1 | 1 | 1 | 0.5 | 0.5 | 1 | 1 | 1 |
| Grewal et al. [59] | 0 | 1 | 1 | 1 | 1 | 1 | 0 | 0.5 | 0 | 0.5 | 0 | 0.5 | 1 | 1 | 0.5 | 0 | 1 | 1 | 0 | 1 | 0 | 1 | 0.5 | 1 | 1 | 1 | 1 | 1 | 1 | 1 |
| Grewal et al. [60] | 1 | 1 | 1 | 1 | 1 | 1 | 0.5 | 0.5 | 0.5 | 1 | 0 | 0.5 | 1 | 1 | 1 | 1 | 0.5 | 1 | 1 | 1 | 0.5 | 0.5 | 0.5 | 1 | 1 | 1 | 1 | 1 | 1 | 0.5 |
| Guo et al. [61] | 1 | 1 | 1 | 1 | 1 | 1 | 0.5 | 0.5 | 1 | 1 | 1 | 1 | 1 | 1 | 1 | 1 | 1 | 1 | 1 | 1 | 1 | 1 | 1 | 0.5 | 1 | 1 | 1 | 1 | 0 | 0 |
| Halickà et al. [62] | 0.5 | 1 | 0 | 0 | 1 | 1 | 0 | 0.5 | 1 | 1 | 0 | 0.5 | 1 | 1 | 0.5 | 0.5 | 1 | 1 | 1 | 1 | 1 | 1 | 0.5 | 0.5 | 0.5 | 0.5 | 1 | 1 | 1 | 1 |
| Heebner et al. [63] | 0.5 | 1 | 1 | 0.5 | 1 | 1 | 1 | 1 | 1 | 1 | 1 | 1 | 1 | 1 | 1 | 0.5 | 1 | 1 | 0.5 | 1 | 0.5 | 1 | 1 | 1 | 1 | 1 | 1 | 1 | 0.5 | 0 |
| Hejda et al. [64] | 1 | 1 | 1 | 1 | 0.5 | 0.5 | 0 | 0 | 1 | 1 | 1 | 1 | 1 | 1 | 1 | 1 | 1 | 1 | 1 | 1 | 1 | 1 | 1 | 1 | 1 | 1 | 1 | 1 | 0 | 0 |
| Hou et al. [65] | 1 | 1 | 1 | 1 | 1 | 0.5 | 1 | 1 | 1 | 1 | 1 | 0.5 | 1 | 1 | 1 | 0.5 | 1 | 1 | 0.5 | 0.5 | 0.5 | 0.5 | 1 | 1 | 1 | 1 | 1 | 1 | 0 | 0 |
| Hsieh et al. [66] | 1 | 1 | 1 | 1 | 1 | 1 | 1 | 1 | 1 | 1 | 1 | 1 | 0.5 | 1 | 1 | 1 | 1 | 1 | 1 | 1 | 0.5 | 1 | 1 | 1 | 1 | 1 | 1 | 1 | 1 | 0.5 |
| King et al. [67] | 1 | 1 | 0.5 | 1 | 1 | 1 | 1 | 1 | 1 | 1 | 1 | 1 | 1 | 1 | 0.5 | 1 | 1 | 1 | 0.5 | 1 | 1 | 0.5 | 1 | 1 | 0.5 | 1 | 1 | 1 | 0 | 0 |
| King et al. [68] | 1 | 1 | 1 | 1 | 1 | 1 | 1 | 0.5 | 1 | 1 | 1 | 1 | 1 | 1 | 1 | 1 | 1 | 1 | 1 | 1 | 1 | 1 | 1 | 1 | 1 | 1 | 1 | 1 | 1 | 1 |

**Table S1.** Results of the quality assessment conducted by raters on the articles included in the review. (*Continued*)

| **First Author (Reference)** | **1** | | **2** | | **3** | | **4** | | **5** | | **6** | | **7** | | **8** | | **9** | | **10** | | **11** | | **12** | | **13** | | **14** | | **15** | |
| --- | --- | --- | --- | --- | --- | --- | --- | --- | --- | --- | --- | --- | --- | --- | --- | --- | --- | --- | --- | --- | --- | --- | --- | --- | --- | --- | --- | --- | --- | --- |
|  | **R1** | **R2** | **R1** | **R2** | **R1** | **R2** | **R1** | **R2** | **R1** | **R2** | **R1** | **R2** | **R1** | **R2** | **R1** | **R2** | **R1** | **R2** | **R1** | **R2** | **R1** | **R2** | **R1** | **R2** | **R1** | **R2** | **R1** | **R2** | **R1** | **R2** |
| Lipsmeier et al. [69] | 1 | 1 | 1 | 1 | 1 | 1 | 0 | 1 | 1 | 1 | 0.5 | 1 | 1 | 1 | 1 | 1 | 1 | 1 | 1 | 1 | 1 | 1 | 1 | 1 | 1 | 1 | 0 | 1 | 1 | 1 |
| Mancini et al.  [70] | 1 | 1 | 1 | 1 | 1 | 1 | 1 | 1 | 1 | 1 | 1 | 1 | 1 | 1 | 1 | 1 | 1 | 1 | 1 | 1 | 1 | 1 | 1 | 1 | 1 | 1 | 1 | 1 | 1 | 0 |
| Mancini et al. [71] | 1 | 1 | 1 | 1 | 1 | 1 | 1 | 1 | 1 | 1 | 1 | 1 | 1 | 1 | 1 | 1 | 1 | 1 | 1 | 1 | 1 | 0.5 | 1 | 1 | 1 | 1 | 1 | 1 | 1 | 0 |
| Matheron et al. [72] | 1 | 1 | 1 | 0.5 | 1 | 1 | 1 | 0.5 | 1 | 1 | 1 | 0.5 | 1 | 1 | 1 | 0.5 | 1 | 1 | 1 | 0.5 | 1 | 1 | 1 | 1 | 1 | 1 | 1 | 0 | 1 | 1 |
| Melecky et al. [73] | 1 | 0.5 | 1 | 0.5 | 1 | 1 | 0.5 | 1 | 1 | 1 | 1 | 1 | 1 | 1 | 1 | 0.5 | 1 | 1 | 0.5 | 1 | 1 | 1 | 1 | 0.5 | 1 | 1 | 1 | 1 | 0.5 | 0 |
| Mellone et al. [74] | 1 | 1 | 1 | 1 | 1 | 0 | 1 | 1 | 1 | 1 | 1 | 1 | 1 | 1 | 1 | 1 | 1 | 1 | 1 | 1 | 1 | 1 | 1 | 1 | 1 | 1 | 1 | 1 | 1 | 1 |
| Nguyen et al. [75] | 0.5 | 0.5 | 1 | 1 | 1 | 0 | 1 | 0.5 | 1 | 1 | 0.5 | 0.5 | 1 | 1 | 1 | 0.5 | 1 | 1 | 0.5 | 1 | 0.5 | 1 | 0.5 | 0.5 | 0.5 | 1 | 0.5 | 0 | 1 | 1 |
| Ozinga et al. [76] | 1 | 1 | 1 | 1 | 1 | 1 | 1 | 0.5 | 1 | 1 | 0 | 1 | 1 | 1 | 1 | 1 | 1 | 1 | 1 | 1 | 0.5 | 0.5 | 1 | 1 | 1 | 1 | 1 | 1 | 1 | 0.5 |
| Palmerini et al. [77] | 1 | 1 | 1 | 1 | 1 | 1 | 1 | 1 | 1 | 1 | 1 | 1 | 1 | 1 | 1 | 1 | 1 | 1 | 1 | 1 | 1 | 1 | 1 | 1 | 1 | 1 | 1 | 1 | 1 | 1 |
| Park et al.  [78] | 1 | 1 | 1 | 1 | 1 | 1 | 1 | 1 | 1 | 1 | 1 | 0.5 | 1 | 1 | 1 | 1 | 1 | 1 | 1 | 1 | 1 | 1 | 1 | 1 | 1 | 1 | 1 | 1 | 1 | 1 |
| Rocchi et al. [79] | 1 | 1 | 1 | 1 | 1 | 1 | 1 | 0.5 | 1 | 1 | 0.5 | 0.5 | 1 | 1 | 1 | 1 | 1 | 1 | 1 | 1 | 1 | 1 | 1 | 1 | 1 | 1 | 1 | 1 | 1 | 1 |
| Rouis et al. [80] | 1 | 1 | 1 | 1 | 0 | 0 | 1 | 0 | 1 | 1 | 0.5 | 0.5 | 1 | 1 | 1 | 1 | 1 | 1 | 0.5 | 0.5 | 0.5 | 0.5 | 1 | 0.5 | 1 | 1 | 1 | 1 | 0.5 | 0.5 |
| Saunders et al. [81] | 1 | 1 | 0 | 0 | 1 | 1 | 0.5 | 0.5 | 1 | 1 | 1 | 1 | 1 | 1 | 1 | 1 | 1 | 1 | 0.5 | 0.5 | 1 | 1 | 1 | 1 | 1 | 1 | 1 | 1 | 1 | 1 |
| Solomon et al.  [82] | 1 | 1 | 1 | 1 | 1 | 1 | 1 | 1 | 1 | 0 | 1 | 0.5 | 1 | 1 | 1 | 1 | 1 | 0 | 1 | 1 | 1 | 1 | 1 | 1 | 1 | 1 | 0 | 0 | 1 | 1 |
| Spain et al. [83] | 1 | 1 | 1 | 1 | 1 | 1 | 1 | 1 | 1 | 1 | 1 | 0.5 | 1 | 1 | 1 | 1 | 1 | 1 | 1 | 1 | 1 | 1 | 1 | 1 | 1 | 1 | 1 | 1 | 1 | 0.5 |

**Table S1.** Results of the quality assessment conducted by raters on the articles included in the review. (*Continued*)

| **First Author (Reference)** | **1** | | **2** | | **3** | | **4** | | **5** | | **6** | | **7** | | **8** | | **9** | | **10** | | **11** | | **12** | | **13** | | **14** | | **15** | |
| --- | --- | --- | --- | --- | --- | --- | --- | --- | --- | --- | --- | --- | --- | --- | --- | --- | --- | --- | --- | --- | --- | --- | --- | --- | --- | --- | --- | --- | --- | --- |
|  | **R1** | **R2** | **R1** | **R2** | **R1** | **R2** | **R1** | **R2** | **R1** | **R2** | **R1** | **R2** | **R1** | **R2** | **R1** | **R2** | **R1** | **R2** | **R1** | **R2** | **R1** | **R2** | **R1** | **R2** | **R1** | **R2** | **R1** | **R2** | **R1** | **R2** |
| Toosizadeh et al. [84] | 1 | 1 | 1 | 1 | 1 | 1 | 0 | 0.5 | 1 | 1 | 0.5 | 1 | 1 | 1 | 1 | 1 | 1 | 1 | 1 | 1 | 1 | 1 | 1 | 1 | 1 | 1 | 1 | 1 | 1 | 0.5 |
| Whitney et al. [85] | 1 | 1 | 1 | 1 | 1 | 1 | 1 | 1 | 1 | 1 | 1 | 1 | 1 | 1 | 1 | 1 | 0 | 0 | 1 | 1 | 1 | 1 | 1 | 1 | 1 | 1 | 1 | 1 | 1 | 1 |
| Zhou et al. [86] | 0 | 0 | 0 | 0.5 | 1 | 1 | 0.5 | 0.5 | 0.5 | 1 | 0 | 0 | 0.5 | 1 | 1 | 1 | 0.5 | 0 | 1 | 1 | 1 | 0.5 | 1 | 1 | 1 | 1 | 1 | 1 | 1 | 1 |

**R1**: Rater 1; **R2**: Rater 2.
