# Supplementary material for: Wearable Inertial Sensors to Assess Standing Balance: A Systematic Review
Source: Sensors (Basel). 2019 Sep 20;19(19):4075. doi: 10.3390/s19194075 (PMC6806601; doi:10.3390/s19194075)
Supplement: Supplementary file 1 [file sensors-19-04075-s001.zip › Supplementary_S2.docx]

**Table S2.** Results of the quality assessment conducted by raters on the articles not included in the review.

| **First Author (Reference)** | **1** | | **2** | | **3** | | **4** | | **5** | | **6** | | **7** | | **8** | | **9** | | **10** | | **11** | | **12** | | **13** | | **14** | | **15** | |
| --- | --- | --- | --- | --- | --- | --- | --- | --- | --- | --- | --- | --- | --- | --- | --- | --- | --- | --- | --- | --- | --- | --- | --- | --- | --- | --- | --- | --- | --- | --- |
|  | **R1** | **R2** | **R1** | **R2** | **R1** | **R2** | **R1** | **R2** | **R1** | **R2** | **R1** | **R2** | **R1** | **R2** | **R1** | **R2** | **R1** | **R2** | **R1** | **R2** | **R1** | **R2** | **R1** | **R2** | **R1** | **R2** | **R1** | **R2** | **R1** | **R2** |
| Abe et al.  [1] | 0.5 | 0.5 | 0.5 | 0.5 | 1 | 1 | 1 | 1 | 1 | 1 | 0.5 | 1 | 0 | 1 | 0 | 0.5 | 1 | 1 | 1 | 1 | 0 | 0.5 | 0.5 | 1 | 0 | 1 | 0 | 1 | 0 | 1 |
| Afzal et al.  [2] | 0 | 1 | 1 | 0.5 | 0 | 1 | 0 | 0 | 1 | 1 | 0 | 0.5 | 1 | 1 | 0.5 | 0 | 0.5 | 1 | 0 | 1 | 0 | 1 | 0 | 0 | 1 | 0 | 1 | 0 | 0 | 0 |
| Afzal et al.  [3] | 0 | 0 | 0 | 0 | 0 | 0 | 0 | 0 | 0 | 0 | 0 | 0 | 0.5 | 0 | 0 | 0.5 | 0 | 0 | 0 | 0.5 | 0 | 0.5 | 0 | 0.5 | 0 | 0 | 1 | 1 | 0 | 0 |
| Alessandrini et al. [4] | 1 | 1 | 1 | 1 | 1 | 1 | 0 | 0 | 1 | 1 | 0 | 0 | 1 | 1 | 1 | 1 | 1 | 1 | 0 | 1 | 0 | 0 | 0 | 0.5 | 1 | 1 | 1 | 0.5 | 0 | 0 |
| Alsubaie et al. [5] | 0.5 | 1 | 1 | 1 | 1 | 1 | 1 | 1 | 0.5 | 1 | 0.5 | 0.5 | 0.5 | 0.5 | 0.5 | 0.5 | 0.5 | 0 | 1 | 0.5 | 0.5 | 0.5 | 0 | 0 | 1 | 0.5 | 0 | 0 | 0.5 | 0.5 |
| Armstrong et al. [6] | 0 | 0.5 | 0 | 0 | 1 | 0 | 0.5 | 0 | 1 | 1 | 0.5 | 1 | 1 | 1 | 0.5 | 0 | 1 | 1 | 0.5 | 1 | 0 | 0 | 0.5 | 0.5 | 0.5 | 0.5 | 1 | 1 | 0 | 0 |
| Badura et al.  [7] | 1 | 0.5 | 1 | 1 | 1 | 1 | 0 | 0.5 | 1 | 1 | 0 | 0.5 | 1 | 1 | 1 | 1 | 1 | 1 | 0 | 1 | 0 | 1 | 0 | 0 | 0 | 0 | 0 | 0 | 1 | 1 |
| Barbado et al. [8] | 1 | 1 | 1 | 1 | 1 | 1 | 1 | 1 | 1 | 1 | 1 | 1 | 0.5 | 0 | 1 | 0 | 1 | 0 | 0.5 | 0 | 1 | 0 | 1 | 0 | 1 | 0 | 1 | 0 | 1 | 1 |
| Bertolotti et al. [9] | 0.5 | 0.5 | 1 | 0.5 | 0 | 0 | 0 | 0.5 | 1 | 1 | 0 | 0 | 1 | 1 | 1 | 0.5 | 1 | 1 | 0 | 1 | 0.5 | 0.5 | 0 | 0 | 0 | 0 | 0 | 0 | 0 | 0 |
| Bonan et al.  [10] | 0 | 0.5 | 0.5 | 0.5 | 1 | 1 | 0.5 | 1 | 1 | 1 | 0 | 0.5 | 1 | 1 | 0 | 0 | 1 | 0.5 | 0 | 0 | 0 | 0 | 0 | 1 | 0.5 | 1 | 0.5 | 1 | 1 | 0.5 |
| Brown et al.  [11] | 1 | 1 | 1 | 1 | 1 | 1 | 1 | 1 | 1 | 1 | 0.5 | 0.5 | 1 | 1 | 1 | 0.5 | 1 | 1 | 1 | 0.5 | 1 | 1 | 1 | 1 | 1 | 1 | 1 | 1 | 1 | 1 |
| Cohen et al.  [12] | 0 | 0 | 0.5 | 0 | 0.5 | 1 | 1 | 0 | 0 | 0.5 | 0 | 0 | 0.5 | 0.5 | 0.5 | 0.5 | 0 | 0 | 0 | 0.5 | 0 | 0.5 | 0 | 0 | 1 | 1 | 1 | 1 | 0.5 | 1 |
| Frames et al.  [13] | 0 | 0 | 0 | 0.5 | 0 | 0 | 0 | 0.5 | 1 | 1 | 0 | 0 | 1 | 1 | 0 | 0.5 | 1 | 0.5 | 0 | 1 | 0 | 0 | 0.5 | 0.5 | 0.5 | 0.5 | 1 | 0.5 | 1 | 1 |
| Franco et al.  [14] | 0 | 0 | 1 | 1 | 0.5 | 1 | 0 | 0 | 1 | 1 | 0 | 0 | 1 | 1 | 0.5 | 0 | 1 | 1 | 1 | 0 | 1 | 0.5 | 0 | 0.5 | 1 | 1 | 0 | 0 | 1 | 1 |

**Table S2.** Results of the quality assessment conducted by raters on the articles not included in the review. (*Continued*)

| **First Author (Reference)** | **1** | | **2** | | **3** | | **4** | | **5** | | **6** | | **7** | | **8** | | **9** | | **10** | | **11** | | **12** | | **13** | | **14** | | **15** | |
| --- | --- | --- | --- | --- | --- | --- | --- | --- | --- | --- | --- | --- | --- | --- | --- | --- | --- | --- | --- | --- | --- | --- | --- | --- | --- | --- | --- | --- | --- | --- |
|  | **R1** | **R2** | **R1** | **R2** | **R1** | **R2** | **R1** | **R2** | **R1** | **R2** | **R1** | **R2** | **R1** | **R2** | **R1** | **R2** | **R1** | **R2** | **R1** | **R2** | **R1** | **R2** | **R1** | **R2** | **R1** | **R2** | **R1** | **R2** | **R1** | **R2** |
| Greene et al.  [15] | 0 | 0 | 0.5 | 0 | 0 | 0 | 0 | 0.5 | 1 | 1 | 0 | 0.5 | 1 | 1 | 0 | 0 | 1 | 1 | 0 | 0.5 | 0 | 0 | 0 | 0 | 0 | 0.5 | 0 | 0 | 1 | 0.5 |
| Han et al.  [16] | 1 | 1 | 1 | 1 | 1 | 1 | 0 | 0 | 1 | 1 | 0 | 0 | 1 | 1 | 0 | 0 | 1 | 1 | 0 | 0 | 0 | 0 | 0 | 0 | 0 | 0 | 1 | 1 | 1 | 1 |
| Huisinga et al. [17] | 0 | 0.5 | 0 | 0 | 0 | 0 | 0 | 0 | 1 | 1 | 0 | 0.5 | 1 | 1 | 0.5 | 0.5 | 1 | 1 | 0.5 | 0.5 | 1 | 1 | 0 | 0.5 | 1 | 1 | 1 | 1 | 1 | 1 |
| Kang et al.  [18] | 1 | 1 | 1 | 1 | 1 | 0 | 0 | 0 | 1 | 1 | 0 | 1 | 1 | 1 | 1 | 0 | 1 | 1 | 0 | 0.5 | 0 | 0 | 1 | 1 | 1 | 1 | 1 | 1 | 0 | 0 |
| Maetzler et al. [19] | 0 | 1 | 0.5 | 1 | 0 | 1 | 0 | 1 | 1 | 1 | 0.5 | 0.5 | 0 | 1 | 0.5 | 1 | 1 | 1 | 0 | 1 | 0 | 0.5 | 0 | 1 | 0.5 | 1 | 1 | 1 | 0 | 0 |
| McGregor et al. [20] | 0 | 0 | 0.5 | 0 | 0.5 | 1 | 1 | 1 | 1 | 1 | 0 | 0.5 | 1 | 1 | 0.5 | 0.5 | 1 | 1 | 0 | 0 | 0 | 0 | 0 | 0.5 | 0.5 | 0.5 | 1 | 1 | 0 | 0 |
| Mendez et al. [21] | 0 | 0.5 | 1 | 1 | 0.5 | 0.5 | 0.5 | 1 | 1 | 1 | 0 | 0.5 | 1 | 1 | 0 | 0.5 | 1 | 1 | 0 | 0 | 0 | 1 | 0 | 0 | 0.5 | 0.5 | 1 | 1 | 0.5 | 0 |
| Mulavara et al. [22] | 0 | 0 | 0.5 | 0.5 | 1 | 1 | 0.5 | 0.5 | 1 | 1 | 0.5 | 0.5 | 0.5 | 1 | 0.5 | 0.5 | 0 | 1 | 0.5 | 0.5 | 0.5 | 0 | 0.5 | 0.5 | 0.5 | 1 | 0 | 0 | 0 | 0 |
| Simila et al.  [23] | 1 | 1 | 1 | 1 | 1 | 1 | 0 | 0 | 1 | 0 | 0 | 0 | 1 | 0 | 1 | 0 | 1 | 1 | 0 | 0 | 0 | 0 | 1 | 1 | 0 | 0 | 0 | 0 | 1 | 1 |
| Steinberg et al. [24] | 0 | 0.5 | 0 | 0 | 1 | 1 | 0 | 0.5 | 1 | 1 | 0 | 0 | 1 | 1 | 0 | 0 | 1 | 1 | 0 | 0.5 | 0 | 0 | 0 | 0 | 1 | 1 | 1 | 1 | 1 | 1 |
| Toosizadeh et al. [25] | 0 | 0.5 | 0 | 0 | 0.5 | 0.5 | 0 | 0 | 1 | 1 | 0 | 0.5 | 1 | 1 | 0 | 0 | 1 | 1 | 0 | 0 | 0 | 0.5 | 0 | 0.5 | 1 | 1 | 1 | 1 | 0 | 0 |
| Yalla et al.  [26] | 0 | 1 | 1 | 1 | 1 | 0.5 | 0 | 0.5 | 1 | 1 | 0 | 0 | 1 | 1 | 0 | 1 | 1 | 0.5 | 0.5 | 1 | 0 | 0.5 | 0.5 | 1 | 0 | 1 | 0 | 1 | 0 | 1 |

**R1**: Rater 1; **R2**: Rater 2.

References

1. Abe, Y.; Sakamoto, M.; Nakazawa, R.; Shirakura, K. Relationship between joint motion and acceleration during single-leg standing in healthy male adults. *J. Phys. Ther. Sci.* **2015**, *27*, 1251–1256.

2. Afzal, M.R.; Oh, M.-K.; Choi, H.Y.; Yoon, J. A novel balance training system using multimodal biofeedback. *Biomed. Eng. Online* **2016**, *15*, 42.

3. Afzal, M.R.; Byun, H.-Y.; Oh, M.-K.; Yoon, J. Effects of kinesthetic haptic feedback on standing stability of young healthy subjects and stroke patients. *J. Neuroeng. Rehabil.* **2015**, *12*, 27.

4. Alessandrini, M.; Micarelli, A.; Viziano, A.; Pavone, I.; Costantini, G.; Casali, D.; Paolizzo, F.; Saggio, G. Body-worn triaxial accelerometer coherence and reliability related to static posturography in unilateral vestibular failure. *Acta Otorhinolaryngol. Ital.* **2017**, *37*, 231–236.

5. Alsubaie, S.F.; Whitney, S.L.; Furman, J.M.; Marchetti, G.F.; Sienko, K.H.; Sparto, P.J. Reliability of Postural Sway Measures of Standing Balance Tasks. *J. Appl. Biomech.* **2018**, 1–23.

6. Jeffrey Armstrong, W.; McGregor, S.J.; Yaggie, J.A.; Bailey, J.J.; Johnson, S.M.; Goin, A.M.; Kelly, S.R. Reliability of mechanomyography and triaxial accelerometry in the assessment of balance. *J. Electromyogr. Kinesiol.* **2010**, *20*, 726–731.

7. Badura, P. Accelerometric signals in automatic balance assessment. *Comput. Med. Imaging Graph.* **2015**, *46 Pt 2*, 169–177.

8. Barbado, D.; Irles-Vidal, B.; Prat-Luri, A.; Garcia-Vaquero, M.P.; Vera-Garcia, F.J. Training intensity quantification of core stability exercises based on a smartphone accelerometer. *PLoS One* **2018**, *13*, e0208262.

9. Bertolotti, G.M.; Cristiani, A.M.; Colagiorgio, P.; Romano, F.; Bassani, E.; Caramia, N.; Ramat, S. A Wearable and Modular Inertial Unit for Measuring Limb Movements and Balance Control Abilities. *IEEE Sens. J.* **2016**, *16*, 790–797.

10. Bonan, I. V; Marquer, A.; Eskiizmirliler, S.; Yelnik, A.P.; Vidal, P.-P. Sensory reweighting in controls and stroke patients. *Clin. Neurophysiol.* **2013**, *124*,
713–722.

11. Brown, H.J.; Siegmund, G.P.; Guskiewicz, K.M.; Van Den Doel, K.; Cretu, E.; Blouin, J.S. Development and validation of an objective balance error scoring system 2013. Available online: https://www.ncbi.nlm.nih.gov/pubmed/24500539 (accessed on 29 July 2019).

12. Cohen, H.S.; Mulavara, A.P.; Peters, B.T.; Sangi-Haghpeykar, H.; Bloomberg, J.J. Standing balance tests for screening people with vestibular impairments. *Laryngoscope* **2014**, *124*, 545–550.

13. Frames, C.W.; Soangra, R.; Lockhart, T.E.; Lach, J.; Ha, D.S.; Roberto, K.A.; Lieberman, A. Dynamical Properties of Postural Control in Obese Community-Dwelling Older Adults (dagger). *Sensors* **2018**, *18*.

14. Franco, C.; Fleury, A.; Gumery, P.Y.; Diot, B.; Demongeot, J.; Vuillerme, N. iBalance-ABF: a smartphone-based audio-biofeedback balance system. *IEEE Trans. Biomed. Eng.* **2013**, *60*, 211–215.

15. Greene, B.R.; Doheny, E.P.; Kenny, R.A.; Caulfield, B. Classification of frailty and falls history using a combination of sensor-based mobility assessments. *Physiol. Meas.* **2014**, *35*, 2053–2066.

16. Han, S.; Lee, D.; Lee, S. A study on the reliability of measuring dynamic balance ability using a smartphone. *J. Phys. Ther. Sci.* **2016**, *28*, 2515–2518.

17. Huisinga, J.; Mancini, M.; Veys, C.; Spain, R.; Horak, F. Coherence analysis of trunk and leg acceleration reveals altered postural sway strategy during standing in persons with multiple sclerosis. *Hum. Mov. Sci.* **2018**, *58*, 330–336.

18. Kang, D.-W.; Seo, J.-W.; Kim, D.-H.; Yang, S.-T.; Choi, J.-S.; Tack, G.-R. A study on balance assessment according to the levels of difficulty in postural control. *J. Phys. Ther. Sci.* **2016**, *28*, 1832–1835.

19. Maetzler, W.; Mancini, M.; Liepelt-Scarfone, I.; Müller, K.; Becker, C.; van Lummel, R.C.; Ainsworth, E.; Hobert, M.; Streffer, J.; Berg, D.; et al. Impaired Trunk Stability in Individuals at High Risk for Parkinson’s Disease. *PLoS One* **2012**, *7*, e32240.

20. McGregor, S.J.; Armstrong, W.J.; Yaggie, J.A.; Bollt, E.M.; Parshad, R.; Bailey, J.J.; Johnson, S.M.; Goin, A.M.; Kelly, S.R. Lower extremity fatigue increases complexity of postural control during a single-legged stance. *J. Neuroeng. Rehabil.* **2011**, *8*, 43.

21. Martinez-Mendez, R.; Sekine, M.; Tamura, T. Postural sway parameters using a triaxial accelerometer: Comparing elderly and young healthy adults. *Comput. Methods Biomech. Biomed. Engin.* **2012**, *15*, 899–910.

22. Mulavara, A.P.; Fiedler, M.J.; Kofman, I.S.; Wood, S.J.; Serrador, J.M.; Peters, B.; Cohen, H.S.; Reschke, M.F.; Bloomberg, J.J. Improving balance function using vestibular stochastic resonance: optimizing stimulus characteristics. *Exp. brain Res.* **2011**, *210*, 303–312.

23. Similä, H.; Immonen, M.; Ermes, M. Accelerometry-based assessment and detection of early signs of balance deficits. *Comput. Biol. Med.* **2017**, *85*, 25–32.

24. Steinberg, N.; Waddington, G.; Adams, R.; Karin, J.; Tirosh, O. Should Ballet Dancers Vary Postures and Underfoot Surfaces When Practicing Postural Balance? *Motor Control* **2018**, *22*, 45–66.

25. Toosizadeh, N.; Ehsani, H.; Miramonte, M.; Mohler, J. Proprioceptive impairments in high fall risk older adults: the effect of mechanical calf vibration on postural balance. *Biomed. Eng. Online* **2018**, *17*, 51.

26. Yalla, S. V; Crews, R.T.; Fleischer, A.E.; Grewal, G.; Ortiz, J.; Najafi, B. An immediate effect of custom-made ankle foot orthoses on postural stability in older adults. *Clin. Biomech. (Bristol, Avon)* **2014**, *29*, 1081–1088.
